# In vivo killing of primary HIV-infected cells by peripheral-injected early memory–enriched anti-HIV duoCAR T cells

**DOI:** 10.1172/jci.insight.161698

**Published:** 2022-11-08

**Authors:** Kim Anthony-Gonda, Alex Ray, Hang Su, Yuge Wang, Ying Xiong, Danica Lee, Ariele Block, Vanessa Chilunda, Jessica Weiselberg, Lily Zemelko, Yen Y. Wang, Sarah Kleinsorge-Block, Jane S. Reese, Marcos de Lima, Christina Ochsenbauer, John C. Kappes, Dimiter S. Dimitrov, Rimas Orentas, Steven G. Deeks, Rachel L. Rutishauser, Joan W. Berman, Harris Goldstein, Boro Dropulić

**Affiliations:** 1 Caring Cross, Gaithersburg, Maryland, USA.; 2 Lentigen, a Miltenyi Biotec Company, Gaithersburg, Maryland, USA.; 3 Department of Microbiology & Immunology and; 4 Department of Pathology, Albert Einstein College of Medicine, Bronx, New York, USA.; 5 Department of Medicine, UCSF, San Francisco, California, USA.; 6 Stem Cell Transplant Program and Center for Regenerative Medicine, University Hospitals Seidman Cancer Center and Case Western Reserve University School of Medicine, Cleveland, Ohio, USA.; 7 Department of Medicine, University of Alabama at Birmingham, Birmingham, Alabama, USA.; 8 Birmingham Veterans Affairs Medical Center, Research Service, Birmingham, Alabama, USA.; 9 Center for Antibody Therapeutics, University of Pittsburgh, Pittsburgh, Pennsylvania, USA.; 10 Department of Pediatrics, University of Washington School of Medicine, and Ben Towne Center for Childhood Cancer Research, Seattle Children’s Research lnstitute, Seattle, Washington, USA.; 11 Department of Pediatrics, Albert Einstein College of Medicine, Bronx, New York, USA.

**Keywords:** AIDS/HIV, Therapeutics, Immunotherapy

## Abstract

HIV-specific chimeric antigen receptor–T cell (CAR T cell) therapies are candidates to functionally cure HIV infection in people with HIV (PWH) by eliminating reactivated HIV-infected cells derived from latently infected cells within the HIV reservoir. Paramount to translating such therapeutic candidates successfully into the clinic will require anti-HIV CAR T cells to localize to lymphoid tissues in the body and eliminate reactivated HIV-infected cells such as CD4^+^ T cells and monocytes/macrophages. Here we show that i.v. injected anti-HIV duoCAR T cells, generated using a clinical-grade anti-HIV duoCAR lentiviral vector, localized to the site of active HIV infection in the spleen of humanized mice and eliminated HIV-infected PBMCs. CyTOF analysis of preinfusion duoCAR T cells revealed an early memory phenotype composed predominantly of CCR7^+^ stem cell–like/central memory T cells (T_SCM_/T_CM_) with expression of some effector-like molecules. In addition, we show that anti-HIV duoCAR T cells effectively sense and kill HIV-infected CD4^+^ T cells and monocytes/macrophages. Furthermore, we demonstrate efficient genetic modification of T cells from PWH on suppressive ART into anti-HIV duoCAR T cells that subsequently kill autologous PBMCs superinfected with HIV. These studies support the safety and efficacy of anti-HIV duoCAR T cell therapy in our presently open phase I/IIa clinical trial (NCT04648046).

## Graphical abstract

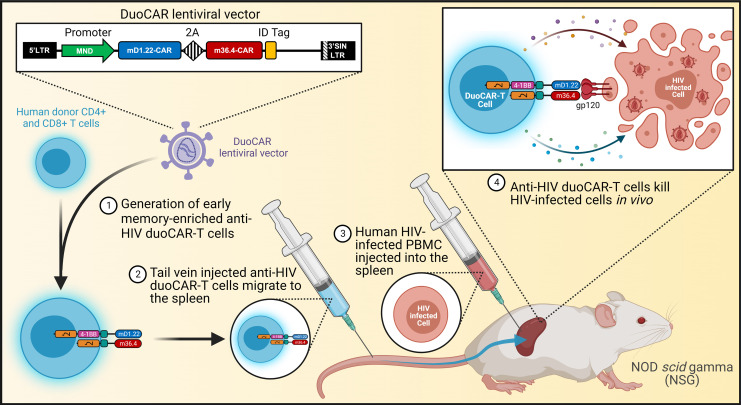

## Introduction

Chimeric antigen receptor–T cell (CAR T cell) therapy has revolutionized the immunotherapy landscape following the development and clinical successes of 4 FDA-approved CAR T cell therapies to treat refractory B cell malignancies (1–4). Recently, 2 people with HIV (PWH) were successfully treated for their diffuse large B cell lymphoma with anti-CD19 CAR T cell therapy, axicabtagene ciloluecel (Yescarta), derived from their autologous T cells. These studies demonstrate that highly functional autologous CAR T cell products from PWH can be generated and safely infused into PWH while on suppressive antiretroviral therapy (ART) to achieve complete cancer remission (5). These successes have reinvigorated previous efforts to translate CAR T cell technology into an arsenal of anti-HIV therapeutics by utilizing potentially novel targeting strategies that focus on overcoming technical hurdles unique to HIV infection (6–16). While these strategies have been the major focus in developing anti-HIV CAR T cell therapy, the safety, efficacy, and feasibility of these therapeutic approaches remain critical factors to assess during preclinical development (17–20).

Clinical trials of first-generation HIV–specific CAR T cells utilizing the CD4 receptor extracellular region to target the HIV envelope (Env) glycoprotein demonstrated that, while they displayed long-term persistence and safety in PWH, they had limited efficacy in controlling HIV (21–23). This was likely due to the reduced activity of first-generation CAR T cells containing only a single anti-HIV targeting domain and TCR ζ signaling motif (Signal 1) and no costimulatory domain (Signal 2), while displaying increased susceptibility to HIV infection due to their overexpression of human CD4 and the HIV coreceptor CCR5 (24). Improved second-generation multitargeting CAR T cells were developed that expressed 1 or more CARs in a single cell engineered with multiple HIV Env–targeting domains combined with additional costimulatory signaling motifs (6, 24). Multitargeting CAR approaches recognize highly conserved sites on the HIV-1 Env spike glycoprotein to prevent immune escape while augmenting resistance of CAR T cells to HIV infection (6, 24). These new approaches amplify the cytotoxic capacity of HIV-specific CAR T cells to target and kill HIV-infected cells while simultaneously protecting the CAR T cells from HIV infection.

Targeting the latent HIV reservoir is paramount for an HIV cure. The latent reservoir is largely composed of memory CD4^+^ T cells and persists, in part, due to clonal expansion of cells harboring intact provirus and continued infection by low levels of virus, despite effective ART (25, 26). Cells of myeloid origin, including monocytes, also contribute to reservoir persistence, as they are resistant to cytotoxic killing, can migrate to distal tissue sites largely inaccessible to CD8^+^ T cells, and can differentiate into long-lived macrophages with self-renewing capacity that may contribute to viral reseeding after ART interruption (27–32). Targeting and eliminating these cell populations during latency or their reactivation is important for developing functional HIV cure strategies.

**Figure 1:**
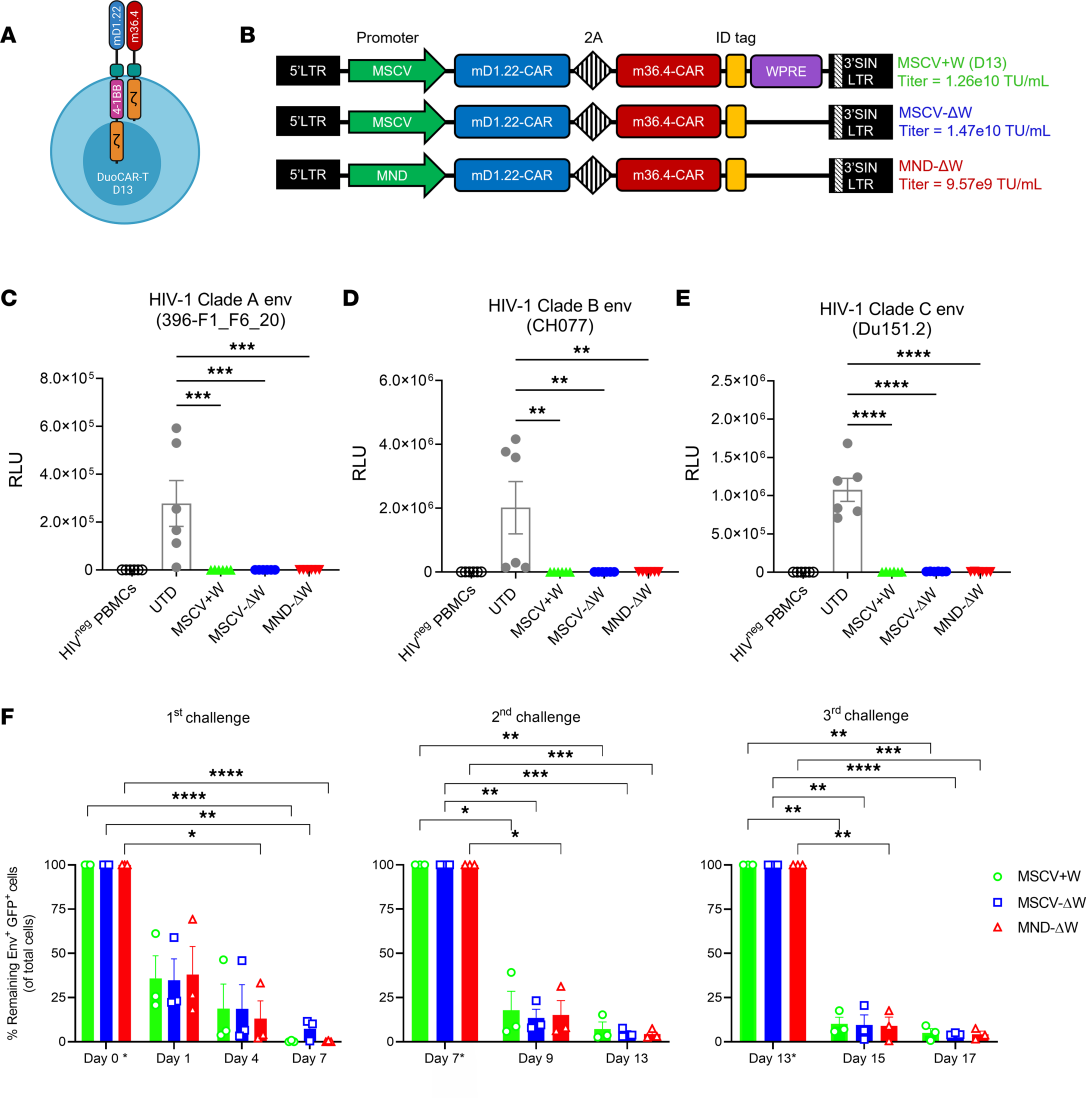
Optimization of anti-HIV duoCAR T cells for clinical translation. (**A**) Illustration of the anti-HIV duoCAR T cell (Created with BioRender.com). (**B**) Schematic of the anti-HIV duoCAR LV constructs evaluated in preclinical studies. MSCV+W is the original anti-HIV duoCAR D13 vector, which contains the MSCV promoter and WPRE. The MSCV+W duoCAR vector was modified for clinical use by excising WPRE (MSCV-ΔW) followed by replacement of the MSCV promoter with the MND promoter (MND-ΔW). A vector identification (ID) tag is engineered upstream of the 3′SIN/LTR for qPCR detection of vector-marked cells. LV titers are indicated to the right of each duoCAR vector in transducing units per mL (TU/mL). (**C**–**E**) In vitro killing efficacy of MSCV+W, MSCV-ΔW, and MND-ΔW duoCAR T cells against autologous PBMCs infected with an HIV-LucR IMC expressing the 396-R1_F6_20 (Clade A), CH077 (Clade B), or Du151.2 (Clade C) HIV-1 Env glycoprotein. Magnitude of HIV-1 infection 7 days after challenge quantified via *Renilla* luciferase (LucR) activity (*y* axis; RLU, relative light units). Data are shown as mean ± SEM of 2 donors tested in triplicate. Statistical analysis performed by 1-way ANOVA followed by Tukey’s multiple-comparison post hoc test. (**F**) Long-term killing efficacy of duoCAR T cells after repeated challenge with Env^+^ GFP^+^ target cells. DuoCAR T cells were challenged with fresh Env^+^ GFP^+^ target cells (E:T ratio = 0.3:1) on Day 0* and subsequently on Day 7* (2nd challenge) and Day 13* (3rd challenge). Asterisks in *y* axis labels indicate date of challenge. Magnitude of duoCAR-mediated killing expressed as percent remaining Env^+^GFP^+^ target cells in the cocultures (*y* axis). Data are shown as mean ± SEM (*n* = 3 donors). Statistical analysis performed by 2-way ANOVA followed by Dunnett’s multiple-comparison post hoc test. ****P < 0.0001, ***P < 0.001, **P < 0.01, and *P < 0.05.

We previously described a unique bicistronic HIV-1–based lentiviral vector (LV) encoding 2 distinct anti-HIV CAR molecules (D13 = mD1.22-CAR + m36.4-CAR), termed duoCAR (Figure 1A), targeting 2 highly conserved HIV-1 gp120 Env glycoprotein epitopes involved in CD4 (mD1.22-CAR) and coreceptor binding (m36.4-CAR) (6). Transduction of T cells with the D13 LV primary conferred T cells with exceptional anti-HIV targeting breadth and potency that enabled them to eliminate HIV-infected cells in humanized mice (6). Here, we extend these studies by developing a D13 duoCAR LV optimized for clinical translation, and we demonstrate its ability to generate anti-HIV duoCAR T cells that display potent in vitro killing of HIV-infected monocytes and CD4^+^ T cells and the in vivo capacity — after i.v. injection — to migrate to the active site of HIV infection in the spleens of humanized mice and potently suppress HIV infection.

## Results

### MND-ΔW duoCAR T cells maintain their breadth and potent anti-HIV killing efficacy in vitro.

To increase the safety of anti-HIV duoCAR T cells for use in clinical trials, we removed the woodchuck posttranscriptional regulatory element (W = WPRE) from the original murine stem cell virus–regulated (MSCV-regulated) D13 LV (MSCV+W) used to generate D13 duoCAR T cells because it contained an intact Protein X open reading frame of unknown function (Figure 1B). We compared the activity of this WPRE^–^ MSCV-regulated duoCAR LV (MSCV-ΔW) to that of a WPRE^–^ duoCAR LV that used a virus-derived and modified MND (short for, myeloproliferative sarcoma virus enhancer, negative-control region deleted dl587rev primer binding site substituted) promoter in lieu of the MSCV promoter (MND-ΔW; Figure 1B). The MND promoter is resistant to transcriptional silencing (33) and drives robust expression of therapeutic genes in hematopoietic-derived cells (34, 35). We evaluated the expression of the 2 distinct anti-HIV CAR molecules, mD1.22-CAR and m36.4-CAR, after transduction of primary human T cells with these duoCAR LV constructs. Expression of the mD1.22-CAR comprising a modified CD4 D1 domain enabled us to gate on CD8^–^ (surrogate for CD4^+^ T cells) and CD8^+^ T cells to quantify duoCAR expression on CD4^+^ and CD8^+^ T cells, respectively. Overall, LV titers (Figure 1B) and transduction efficiency were similar among the 3 anti-HIV duoCAR LV constructs (Supplemental Figure 1; supplemental material available online with this article; https://doi.org/10.1172/jci.insight.161698DS1). The FDA-recommended threshold of < 5 vector copy number (VCN)/transduced cell was achieved using the MND-ΔW duoCAR LV and high CAR transduction efficiency in MND-ΔW duoCAR T cells (Supplemental Figure 2).

We evaluated the capacity of anti-HIV duoCAR T cells generated after transduction with the MSCV+W, MSCV-ΔW, or MND-ΔW LV to eliminate HIV-infected cells using our previously described in vitro CAR T cell killing assay (6). Briefly, we quantitated the reduction in magnitude of the HIV infection after anti-HIV duoCAR T cells were challenged with donor-matched peripheral blood mononuclear cells (PBMCs) infected with an infectious molecular clone (IMC) of HIV that expresses a *Renilla* luciferase reporter and the HIV-1 Env glycoprotein from either Clade A (isolate 396-F1_F6_20), B (isolate CH077), or C (isolate Du151.2) HIV-1 strains (hereafter referred to as HIV-LucR; ref. 36). HIV-LucR IMC efficiently replicate in PBMCs (MOI = 0.0025) and display in vitro replication kinetics similar to their respective nonreporter parental virus when infected at higher MOIs (36). The HIV-1 Env sequences encoded by these HIV-LucR IMCs cover a wide range of the worldwide genetic HIV-1 Env diversity. Anti-HIV duoCAR T cells almost completely suppressed in vitro cellular HIV-1 infection propagated by donor-matched human PBMCs infected with an HIV-LucR IMC (MOI = 1) expressing a Clade A Env (Figure 1C), a Clade B Env (Figure 1D), or a Clade C Env (Figure 1E) glycoprotein.

Next, we compared the impact of the MSCV and MND promoters used to drive duoCAR expression on the long-term in vitro cytotoxicity of anti-HIV duoCAR T cells. Previous studies using CD19-targeted CAR T cells have shown that optimal CAR expression, which may be influenced by the gene promoter, plays an important role in sustained CAR T cell function (37). We challenged the anti-HIV duoCAR T cells 3 times at weekly intervals with HEK293T cells that coexpress the HIV-1 Env glycoprotein and GFP (Env^+^GFP^+^), a surrogate for HIV-infected cells, to determine their ability to persist and durably eliminate Env^+^GFP^+^ target cells. The anti-HIV duoCAR T cells displayed a significant and durable ability to eliminate the Env^+^GFP^+^ target cells (>90% killing activity observed on day 7 [D7], D13, and D17) after repeated challenge during the 17-day coculture period (Figure 1F; gating strategy shown in Supplemental Figure 3). We postulate that loss of Env expression by the Env^+^GFP^+^ target cell population over time in the absence of antibiotic selection may prevent total elimination of these target cells during long-term coculture. Overall, the duoCAR T cells generated by the MND-ΔW duoCAR vector displayed high CAR expression with lower VCNs than duoCAR T cells generated by the MSCV-ΔW duoCAR vector and maintained potent anti-HIV killing activity. Based on these data, the MND-ΔW duoCAR vector was selected for further evaluation in a humanized mouse model of intrasplenic HIV-1 infection.

### Therapeutic efficacy of i.v. injected duoCAR T cells in humanized mice with intrasplenic HIV infection.

We previously evaluated the in vivo anti-HIV efficacy of research-scale CAR T cells in a PBMC-humanized *NOD.Cg-Prkdc^scid^ Il2rg^tm1Wjl^/SzJ* (NSG) mouse model of intrasplenic HIV-1 infection (hu-spl-PBMC-NSG) by intrasplenically coinjecting CAR T cells with HIV-1 spinoculated PBMCs (1 × 10^7^), followed by quantitation of HIV infection within the PBMCs of the spleen 7 or 30 days later (6). Effective treatment of PWH will require migration of i.v. injected CAR T cells to lymphoid and other tissues harboring HIV-infected cells. Therefore, we examined whether i.v. injected vector-optimized anti-HIV duoCAR T cells, manufactured using an automated closed-system device (CliniMACS Prodigy) and clinical trial protocol, could migrate to the mouse spleen in the hu-spl-PBMC-NSG mice and eliminate HIV-infected PBMCs.

**Figure 2:**
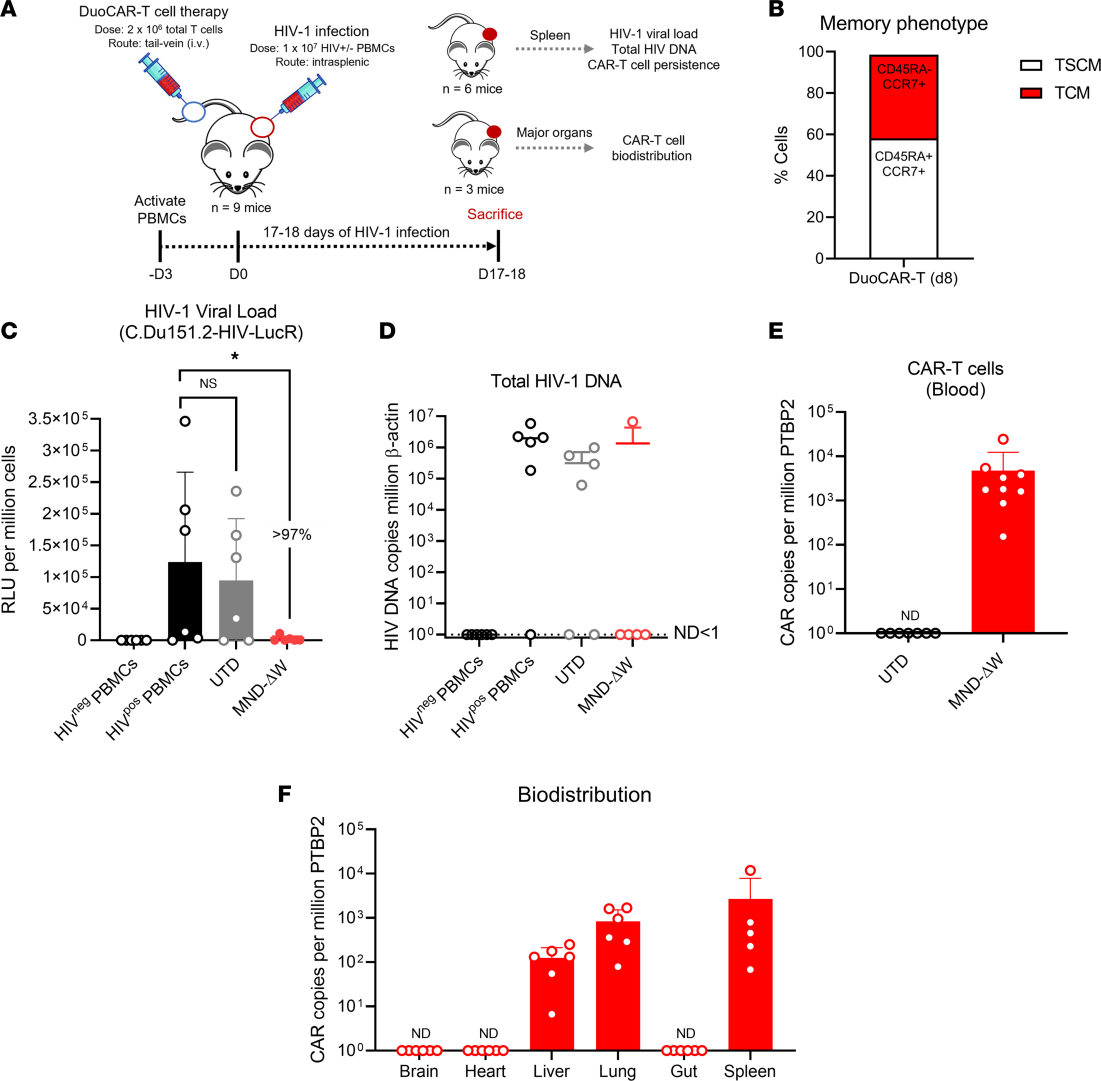
In vivo localization and potent anti-HIV efficacy of i.v.-administered anti-HIV duoCAR T cell therapy in humanized HIV-infected mice. (**A**) Illustration of the hu-spl-PBMC-NSG model of HIV-1 infection. The safety and efficacy of i.v.-administered anti-HIV duoCAR T cell therapy were evaluated in PBMC-humanized NSG mice after 17–18 days of intrasplenic HIV-1 infection. (**B**) T cell memory phenotype of the preinfusion anti-HIV duoCAR T cell product manufactured on the CliniMACS Prodigy device (*n* = 2 donors). T_SCM_ (CD45RA^+^CCR7^+^) and T_CM_ (CD45RA^–^CCR7^+^) cell populations were determined by flow cytometry. T_SCM_ were delineated from T_N_ (naive T cells [CD45RA^+^CCR7^+^CD95^-^]) cells by the presence of CD95^+^ on CD45RA^+^CCR7^+^ cells (gating strategy shown in Supplemental Figure 4). (**C**) Quantification of HIV-1 viral load via *Renilla* luciferase (LucR) activity in the spleens of humanized mice after 17–18 days of HIV-1 infection. (**D**) Quantification of cell-associated total HIV-1 DNA in the spleens of humanized mice after 17–18 days of HIV-1 infection. Results are expressed as HIV-1 Gag DNA copies per 1 million β-actin copies. ND, not detected. One of the mice in the MND-ΔW duoCAR T cell–treated group had insufficient cells for HIV DNA analysis; therefore, only 5 samples were evaluated for this group. (**E** and **F**) Persistence and biodistribution profile of intravenously administered anti-HIV duoCAR T cells in the blood and major organs of humanized mice after 17–18 days of HIV-1 infection. Data are expressed as CAR DNA copies per 1 million polypyrimidine tract binding protein 2 (PTBP2) copies. Data are shown as mean ± SD of samples tested (*n* = 5–9 mice). Statistical analysis was performed by 1-way ANOVA followed by Dunnett’s multiple-comparison post hoc test. *P < 0.05.

Anti-HIV duoCAR T cells (2 × 10^6^ total T cells) were i.v. injected into the tail veins of mice following intrasplenic injection of syngeneic HIV-LucR spinoculated PBMCs (1 × 10^7^ cells) (Figure 2A). Preinfusion duoCAR T cells were enriched for early memory T cells expressing phenotypic markers associated with stem cell memory–like (T_SCM_; defined as CD45RA^+^CCR7^+^CD95^+^; refs. 38, 39) and central memory (T_CM_; defined as CD45RA^–^CCR7^+^) T cells (Figure 2B; gating strategy shown in Supplemental Figure 4). Mice were sacrificed at a single time point (D17 or D18) and analysis of the mouse spleens demonstrated that i.v. treatment with MND-ΔW duoCAR T cells markedly reduced the magnitude of HIV-1 infection in the mice (>97%) as compared with the spleens of untreated mice or mice treated i.v. with untransduced (UTD) control T cells (Figure 2C, P *<* 0.05). The spleens from 5 of 6 untreated mice (HIV^+^ PBMC cohort) and 4 of 6 UTD T cell–treated mice displayed high levels of cell-associated total HIV-1 DNA (averaging ~ 1 × 10^6^ and 1 × 10^5^ copies, respectively) (Figure 2D). Four of 5 mice treated with MND-ΔW duoCAR T cells displayed potent suppression of HIV-1 infection and undetectable levels of cell-associated total HIV-1 DNA in their spleens (Figure 2D). We postulate that the single mouse with nearly undetectable viral loads, but concomitant detectable total HIV DNA, may represent detection of replication-defective HIV by the total HIV DNA quantitative PCR (qPCR) assay. In this case, those cells containing replication-defective HIV may not express sufficient levels of surface Env for CAR-mediated killing.

All of the anti-HIV duoCAR T cell–treated mice survived the duration of the study (17-18 days) and did not display significant body weight changes or atypical behavior. The engraftment and persistence of circulating MND-ΔW duoCAR T cells in mouse blood and organs after i.v. injection was assessed at the study end point (D17 and D18) using a highly sensitive qPCR assay capable of detecting 1 copy/μL/reaction. MND-ΔW duoCAR T cells engrafted and persisted in the peripheral blood of all the HIV-infected mice evaluated 17–18 days after infection (Figure 2E); this also occurred in the spleen (site of HIV infection), lung, and liver tissues but not in the mouse brain, heart, or gut intestinal tissues (Figure 2F). Based on the preclinical data, the MND-ΔW duoCAR vector was selected as the final clinical candidate to generate anti-HIV duoCAR T cell products for further evaluation in preparation for our clinical trial.

### Successful manufacture of T_SCM_/T_CM_ enriched anti-HIV duoCAR T cells from PWH.

CAR T cell products with a T_SCM_ or T_CM_ phenotype are highly desirable, given their propensity for memory formation, self-renewal, persistence, lower potential to induce cytokine release syndrome (CRS), and improved therapeutic efficacy (40). While the central memory bias of ex vivo–expanded T cells is well documented for cancer-specific CAR T cell products (40–43), very little is known about the central memory phenotype of ex vivo expanded anti-HIV CAR T cells from PWH. Such a product can be generated by a cell manufacturing process that is 8 days in duration, shorter than the typical 12- to 14-day process (44). Previously, we have validated a GMP-compliant, 8-day manufacturing process using an automated closed-system device (CliniMACS Prodigy) for our anti-CD19 CAR T cell clinical trials (42). In preparation for our anti-HIV duoCAR clinical trial, we adopted a modified version of the 8-day manufacturing process, which did not use human AB serum or antiretroviral (ARV) drugs to generate anti-HIV duoCAR T cells. We validated the modified process at the manufacturing site that will be used for the clinical trial using apheresis material from ART-suppressed PWH donors transduced with the anti-HIV duoCAR LV (1.03 × 10^10^ TU/mL) selected for clinical trials (MND-ΔW) generated under GMP conditions. Table 1 shows the performance characteristics of the 8-day CAR T cell manufacturing process, which is free of ARV drugs, used to generate anti-HIV duoCAR T cell products from the T cells of 2 ART-suppressed PWH. The 2 PWH donors used in the manufacturing and phenotyping studies were from the UCSF cohort. Overall, duoCAR T cell products exhibited high T cell purity (>98%), viability (>86%), and CAR transduction efficiency in total CD3^+^ T cells (median, 55.58%), CD4^+^ and CD8^+^ T cell subsets (median, 45.46% and 12.22%, respectively), and duoCAR^+^ CD8^+^ T cells (median, 33.29%). The frequency of CD4^+^ T cells was ~3-fold higher than CD8^+^ T cells (median, 77.66% and 22.01%, respectively) in the final products, indicating no impact of HIV infection on CD4^+^ T cells despite using an ARV-free manufacturing process. The product passed all release criteria, and we generated 600–800 million CAR^+^ T cells with a total cell expansion capacity of > 12-fold (>1 × 10^9^ total cells). VCN was below the FDA-recommended 5 copies per transduced cell for both donor products (2.27 and 3.29 VCN per transduced cell for Donor 1 and Donor 2, respectively). We note that it is not unusual that the lower CAR transduction rate on the T cell’s surface (50% as measured by the less sensitive flow cytometry method) may be the result of transduction of multiple copies of vector DNA per transduced cell (as measured by the more sensitive qPCR method) and/or due to transcriptional or posttranscriptional silencing of a subset of integrated vector transgenes. Using VCN reference standards developed by our group (45), we have validated the ability of the VCN qPCR assay to accurately and reproducibly quantify 1, 2, 3, or 4 vector copies in the genome/cell that is within the range of the VCNs observed in our duoCAR T cell products. Notably, we did not detect reactivation of HIV in the duoCAR T cell products manufactured without ARV drugs, as assessed by cell-associated total HIV DNA, HIV-1 p24 antigen, and replication competent lentivirus (RCL) biological (cell-culture) assays. Last, we found no evidence of transgene mobilization, which would be unlikely to occur since we used a self-inactivating–based (SIN-based) duoCAR LV. These results demonstrate that clinical-grade anti-HIV duoCAR T cells products can be safely manufactured from the T cells of PWH using an 8-day ARV drug- and serum-free process.

**Figure 3:**
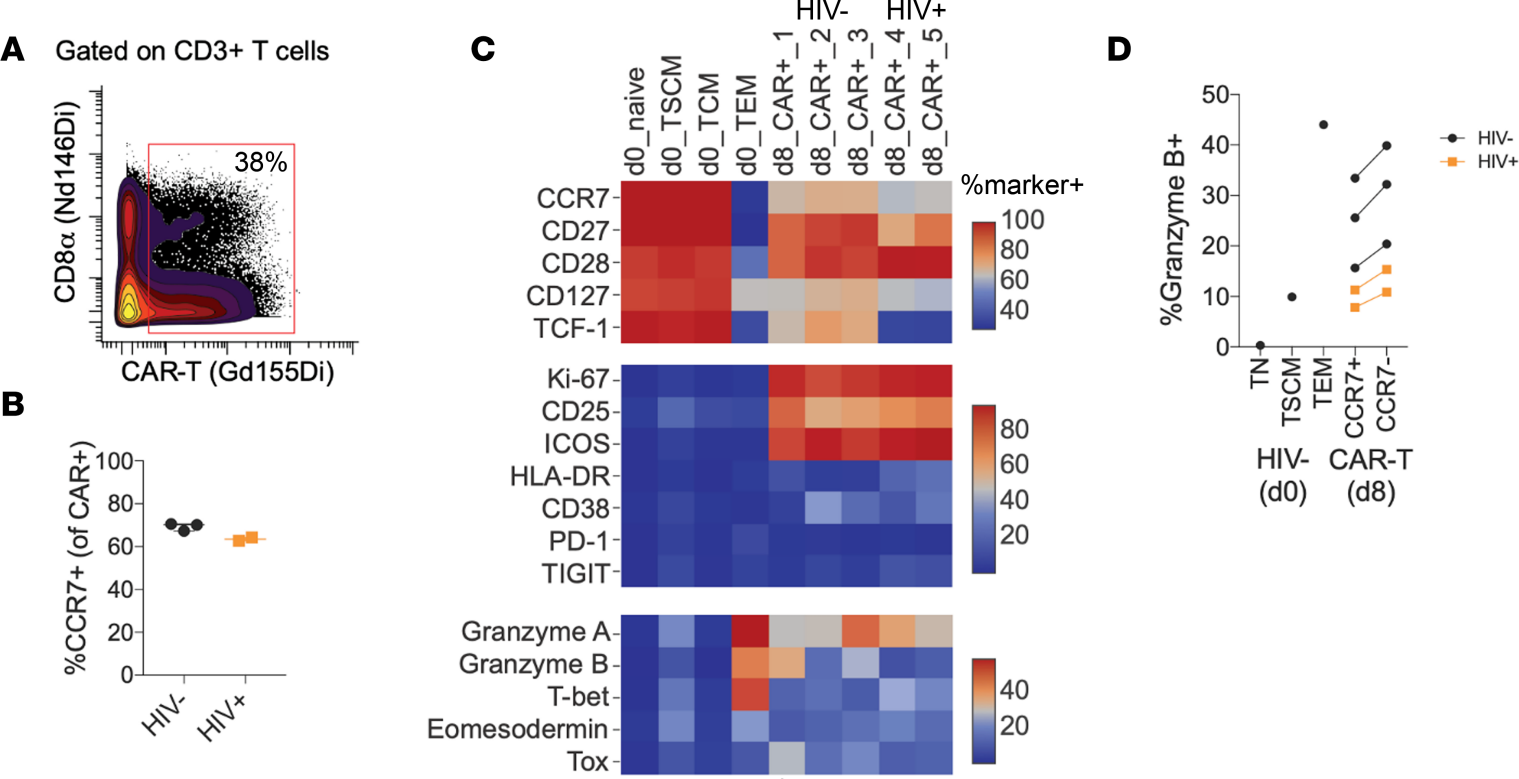
CyTOF analysis of preinfusion anti-HIV duoCAR T cell products reveals a T_CM_/T_SCM_ phenotype primed for lymphoid tissue homing and effector function. (**A**) Gating strategy to identify D8 anti-HIV duoCAR T cells (% CAR^+^ of total CD3^+^ T cells) by mass cytometry staining. (**B**) Frequency of summed T_SCM_ + T_CM_ phenotype (CCR7^+^) duoCAR T cells at D8 among HIV seronegative (HIV^–^, CAR^+^_1, CAR^+^_2, CAR^+^_3; *n* = 3) and seropositive donors (HIV^+^, CAR^+^_4, CAR^+^_5; *n* = 2). (**C**) Heatmap showing the phenotype (scaled by % marker^+^) of D8 duoCAR T cells from the same HIV^–^ or HIV^+^ donors compared with untransduced (D0) T cell subsets from an HIV^–^ donor. (**D**) Expression of Granzyme B in D0 T cell subsets compared with CCR7^+^ or CCR7^–^ subsets from D8 CAR^+^ product. TSCM, stem cell memory; TCM, central memory; TEM, effector memory.

We characterized the differentiation state of clinical-scale duoCAR T cell products from PWH using mass cytometry by TOF (CyTOF) using a metal-conjugated antibody directed against the m36.4-CAR, which reliably detected duoCAR-T^+^ cells (Figure 3A) at similar frequencies compared with flow cytometry using a fluorophore-conjugated antibody (Table 1). Anti-HIV duoCAR T cell products were manufactured from HIV^–^ and ART-suppressed PWH (HIV^+^) donor apheresis material using an equivalent manufacturing process and GMP-grade critical reagents (e.g., TransAct reagent, IL-2 cytokine, and LV duoCAR) used to manufacture duoCAR T cells on the CliniMACS Prodigy. Among the 5 donors tested (*n* = 3, HIV^–^; *n* = 2, HIV^+^), we confirmed that anti-HIV duoCAR T cells were predominately enriched for CCR7^+^ T_SCM_ and T_CM_ cells (Figure 3B), which is an important chemokine receptor involved in directing T cells to migrate into secondary lymphoid organs (e.g., lymph nodes and spleen). DuoCAR^+^ T cells generated from uninfected individuals or PWH expressed high levels of T cell memory markers (CD27, CD28, and CD127; Figure 3C), markers of T cell proliferation (Ki-67) and activation (particularly CD25 and ICOS — and, to some extent, HLA-DR and CD38; Figure 3C). The duoCAR T cells derived from HIV^–^ individuals had a higher frequency of TCF-1^+^ cells, a marker of T cell stemness (46), and lower HLA-DR expression compared with PWH-derived duoCAR T cells (TCF-1: median 72% versus 34.5%; HLA-DR: median 5% versus 25.5%; Figure 3C). The difference in TCF-1 and HLA-DR expression observed in the duoCAR T cell products is consistent with our previous findings of HIV-specific and bulk CD8^+^ T cells from ART-suppressed PWH (47). Of note, 2 coinhibitory receptors (PD-1 and TIGIT) and Tox, a transcription factor associated with exhaustion, were not substantially elevated in D8 products relative to D0 T_EM_ subset cells (Figure 3C). Cytolytic effector proteins (Granzyme A and B), as well as transcription factors (T-bet and Eomesodermin), involved in T cell effector differentiation were also expressed in the duoCAR T cell products (Figure 3C), with a slightly higher level of Granzyme B expression found in the non-T_SCM_/T_CM_ (i.e., CCR7^–^) subset of duoCAR-T^+^ cells (Figure 3D). These results show that clinical-grade PWH-derived anti-HIV duoCAR T cell products are predominately composed of T_SCM_/T_CM_ cells with some markers of effector differentiation, a preferred phenotype that suggests that duoCAR T cells would traffic to lymphoid tissues, display long-term persistence in the body, and immediately exert effector function upon sensing HIV Env on the surface of productively HIV-infected cells.

### Anti-HIV duoCAR T cells kill HIV-infected monocytes in vitro.

**Figure 4:**
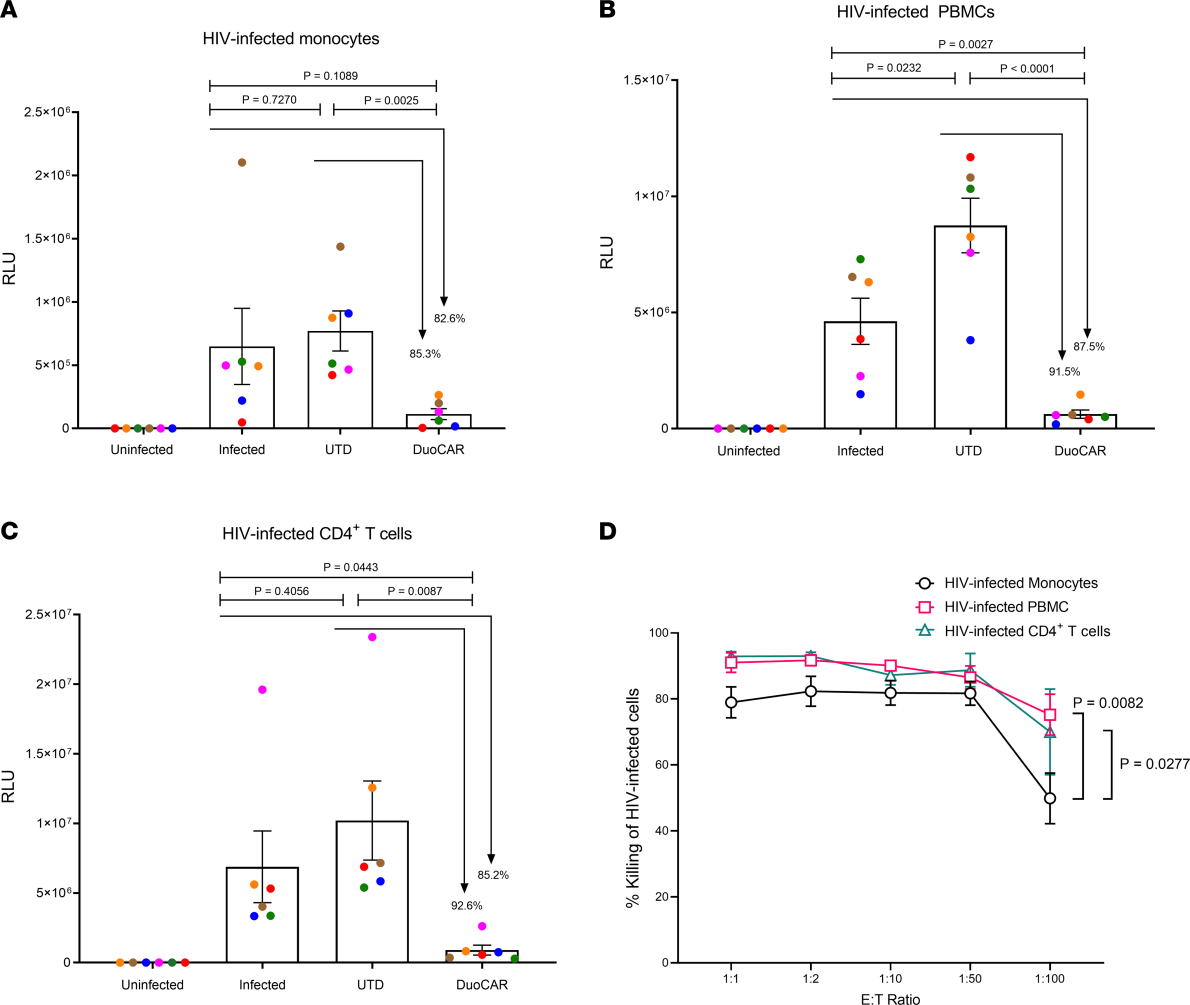
Anti-HIV duoCAR T cells recognize and potently kill HIV-infected monocytes. (**A**–**C**) Purified and matured monocytes (**A**), PBMCs (**B**), or CD4^+^ T cells (**C**) from the same donor were infected with HIV_BaL_-LucR virus for 2 days (monocytes) or 3 days (PBMCs and CD4^+^ T cells). HIV-infected cells were either untreated (infected) or treated with donor-matched untransduced (UTD) T cells or MND-ΔW duoCAR T cells (duoCAR) at an E:T ratio of 1:1 for an additional 3 days. Uninfected cells were used as negative controls in the assay. The magnitude of HIV-1 infection was quantified 3 days after infection by measuring *Renilla* luciferase activity and was expressed as relative light units (RLU). Data are shown as mean ± SEM. The percent HIV-1 suppression is shown above the bar graph for MND-ΔW duoCAR T cells and was calculated relative to infected cells either left untreated or treated with UTD control T cells. The study shown is from 6 independent experiments with cells from 6 different HIV-1 seronegative donors. Statistical analysis was performed by unpaired Student’s *t* test. Significance is considered *P* < 0.05. (**D**) Sensitivity of anti-HIV duoCAR T cell killing against monocytes, PBMCs, and CD4^+^ T cells. Percent killing of HIV-infected cells was calculated relative to UTD control T cells. Data are shown as mean ± SEM. Data show *n* = 4 HIV seronegative donors for all E:T ratios except 1:100, for which data show *n* = 3. Statistical analysis was performed by 2-way ANOVA followed by Tukey’s multiple comparison post hoc test.

In addition to HIV-infected memory CD4^+^ T cells, HIV-infected monocytes/macrophages represent a component of the HIV reservoir in ART-suppressed PWH that persist due to their resistance to killing by cytotoxic lymphocytes (CTLs) (48) and are capable of producing HIV to mediate viral rebound after ART interruption (28). Therefore, we examined the capacity of MND-ΔW duoCAR T cells to kill HIV-infected monocytes as compared with HIV-infected PBMCs and CD4^+^ T cells. PBMCs isolated from HIV-1 seronegative donors, and monocytes and CD4^+^ T cells purified from these PBMCs, were infected with an HIV-LucR IMC expressing an Env glycoprotein derived from the BaL HIV-1 isolate (Group M, Clade) (1 × 10^6^ IU/mL). After 3 days of culture to establish infection, the HIV-infected PBMCs, CD4^+^ T cells, or monocytes (1 × 10^5^ cells/well) were either left untreated or treated with added autologous UTD T cells or anti-HIV duoCAR effector T cells (effector/target [E:T] ratio of 1:1). Three days later, HIV infection was quantified by LucR activity in the cell lysates. As shown in Figure 4, the anti-HIV duoCAR T cells potently suppressed HIV infection in HIV-infected monocytes by 85.3% (Figure 4A), PBMCs by 91.5% (Figure 4B), and CD4^+^ T cells by 92.6% (Figure 4C), as compared with treatment with UTD control T cells. Furthermore, duoCAR T cells exerted a robust killing effect against HIV-infected monocytes across a wide range of E:T ratios tested (1:1 to 1:100), comparable with that of HIV-infected PBMCs and CD4^+^ T cells. At the lowest 1:100 E:T ratio, duoCAR T cells were still able to recognize and kill nearly 50% of HIV-infected monocytes in the coculture and killed > 70% of PBMCs and CD4^+^ T cells, albeit significantly better than monocytes (Figure 4D, P = 0.0082, PBMCs; *P* = 0.0277, CD4^+^ T cells). This result demonstrates the sensitive recognition and robust killing of HIV-infected monocytes, a component of the HIV reservoir, by anti-HIV duoCAR T cells.

### Anti-HIV duoCAR T cells generated from PWH demonstrate potent effector function.

Despite effective control of HIV replication in ART-treated PWH, the chronic activation and inflammation induced by HIV infection compromises the function of CD8^+^ T cells — particularly their cytotoxic capacity (49). Consequently, CAR T cells derived from PWH may display compromised cytotoxic activity, limiting their capacity to control HIV replication in vivo after infusion into PWH. Therefore, we investigated the anti-HIV activity of MND-ΔW duoCAR T cells generated from the T cells of 5 ART-suppressed PWH, including 1 long-term nonprogressor (LTNP) (donor HGLK047) whose clinical and laboratory details are listed in Table 2, which are different PWH donors from the 2 used in Table 1. As shown in Supplemental Table 1 (donors characteristics shown in Table 2), anti-HIV duoCAR T cell products generated at research scale from the 5 PWH donors demonstrated high T cell purity (>98% CD3^+^ T cells), viability (>90% viable T cells), and transduction efficiencies of > 50% for LV-modified CD4^+^ T cells (range, 53%–75%) and > 30% for LV-modified CD8^+^ T cells (range, 30%–66%) with less than 5 vector copies per cell.

**Figure 5:**
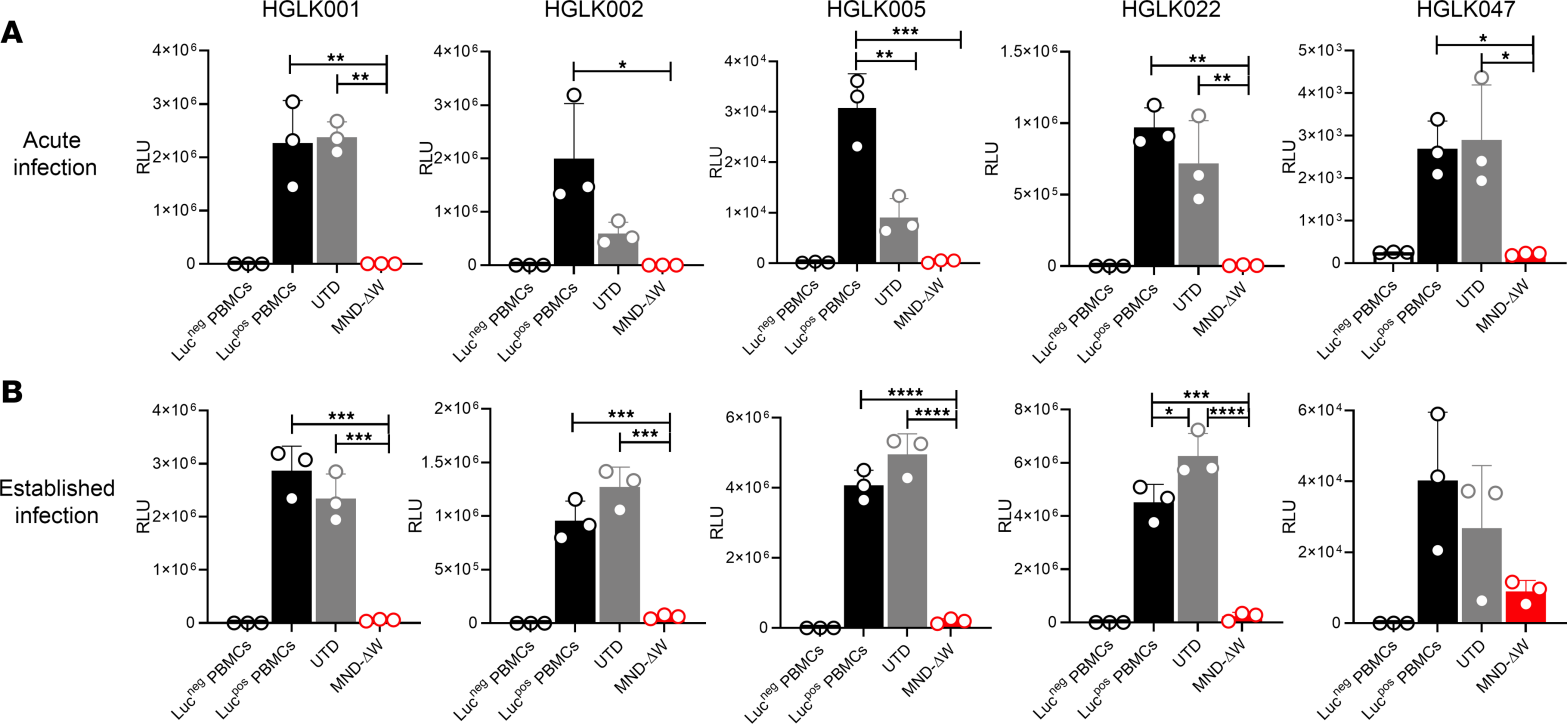
MND-ΔW duoCAR T cells derived from PWH potently kill autologous cells infected with HIV in vitro. (**A** and **B**) In vitro CAR T cell–mediated killing of CD8-depleted PBMCs isolated from PWH with acute or established HIV-1 superinfection (BaL Env, clade B). Each graph represents a different PWH donor, and the donor’s unique identifier is indicated at the top of its respective graph. HGLK001, HGLK002, HGLK005, and HGLK022 donors are PWH who are ART suppressed, with undetectable HIV-1 viral loads at the time of blood collection. Donor HGLK047 is a long-term nonprogressor with an undetectable HIV-1 viral load at the time of blood collection. For acute infection studies, autologous MND-ΔW duoCAR T cells or untransduced (UTD) control T cells were added shortly after spinfection of PBMCs with HIV-LucR (E:T = 1:1) and challenged for 3 days, followed by quantification of HIV-1 infection (LucR activity). For established infection studies, HIV-LucR spinfected PBMCs were cultured for 3 days to establish HIV infection followed by addition of autologous duoCAR T cells or UTD control T cells (E:T = 1:1). The cocultures were challenged for an additional 3 days, followed by quantification of HIV-1 infection (LucR activity). The *y* axis shows the magnitude of the HIV-1 infection as quantified via *Renilla* luciferase (LucR) activity and expressed as relative light units (RLU). Uninfected CD8-depleted PBMCs serve as a negative control for the assay (Luc^–^ PBMCs). CD8-depleted PBMCs superinfected with HIV-LucR serve as a positive control for the assay (Luc^+^ PBMCs). Data are shown as mean ± SD of triplicate sample wells tested. Statistical analysis was performed by 1-way ANOVA, followed by Tukey’s multiple comparison post hoc test; Luc^–^ PBMCs were not included in the statistical analysis. ****P < 0.0001, ***P < 0.001, **P < 0.01, and *P < 0.05.

The functional activity of PWH-derived anti-HIV duoCAR T cells was evaluated utilizing a modified version of our in vitro CAR T cell killing assay against HIV-infected PBMCs. The low frequency of HIV-infected PBMCs in virus-suppressed PWH on long-term suppressive ART required us to superinfect the PBMCs with an HIV-LucR IMC to determine the ability of anti-HIV duoCAR T cells to suppress HIV infection in autologous PBMCs from PWH. CD8^+^ T cell–depleted PBMCs from these donors were superinfected with a HIV-LucR IMC expressing the BaL Env and cocultured with autologous MND-ΔW duoCAR T cells either immediately following the initiation of HIV-LucR infection (Figure 5A, acute infection) or 3 days later, after HIV-LucR infection had been established (Figure 5B, established infection). As compared with HIV-infected PBMCs treated with UTD control T cells, the addition of anti-HIV duoCAR T cells almost completely suppressed acute HIV-infection in all 5 donors and established infection in 4 of the 5 donors, with 1 donor exhibiting less-potent HIV suppression (HGLK047) (Figure 5, A and B). Taken together, our data demonstrate that T cells from PWH can be used to safely generate anti-HIV duoCAR T cell products that have potent anti-HIV activity and are capable of eliminating autologous HIV-infected cells superinfected with HIV.

## Discussion

The persistence of the HIV reservoir despite suppressive ART is a major obstacle to achieving an HIV cure in PWH. Achievement of a functional cure in the absence of ART would require PWH to have circulating immune effector cells resistant to HIV infection that can rapidly eliminate HIV-infected cells emerging from latency in anatomical sites known to harbor the HIV reservoir. Such a therapy would thereby prevent local spread and the subsequent reintroduction and dissemination of systemic HIV infection. In the current study, we focused on preclinical studies to advance our potent anti-HIV duoCAR T cell therapy, which also displays resistance to HIV infection, into clinical trials to determine their capacity to provide a sustained immune response enabling a functional cure for HIV. We created an anti-HIV duoCAR T cell product for clinical translation by removing the WPRE element from the duoCAR LV to increase its safety, and we utilized the MND promoter, which is less prone to transcriptional silencing (MND-ΔW duoCAR) than the MSCV promoter to control CAR transgene expression. We demonstrated that the MND-ΔW duoCAR T cells potently eliminated HIV-infected CD4^+^ T cells and monocytes/macrophages. Furthermore, we showed that i.v. injected MND-ΔW duoCAR T cells migrated to the active site of HIV infection in the spleens of humanized mice and potently suppressed virus replication. Finally, we established that the MND-ΔW duoCAR LV can convert primary human T cells from PWH into duoCAR T cells with potent anti-HIV activity. Collectively, these investigational new drug-enabling studies provide important clinical translational insights that build upon our previous in vitro and in vivo proof-of-concept studies, which have led to the initiation of a first-in-human phase I/IIa study to evaluate the safety and efficacy of anti-HIV duoCAR T cells therapy in PWH.

A major goal of this study was to evaluate the ability of anti-HIV duoCAR T cells to localize to the site of active HIV infection in the spleens of HIV-infected hu-spl-PBMC-NSG mice. We found that i.v. injected anti-HIV duoCAR T cells entered the spleens of humanized mice and significantly suppressed virus infection (>97% HIV suppression, *P* *<* 0.05). This finding is consistent with our previous work, which showed durable and potent in vivo suppression of HIV-1 infection and prevention of CD4^+^ T cell depletion for 30 days in humanized mice treated with anti-HIV duoCAR T cell therapy (6). Expression on the duoCAR T cell surface of CCR7, which directs T cell homing to secondary lymphoid organs and can bind to human and mouse CCL21 (50), may have facilitated the in vivo migration of these cells from the peripheral blood into the infected mice spleens (51), permitting access to HIV-infected PBMCs.

As expected, i.v.-administered duoCAR T cells were also detected in the liver and lung tissues of treated mice, and the biodistribution profile of duoCAR T cells closely resembled that found in humans receiving other CAR T cell immunotherapies currently used to treat B cell malignancies (52, 53). Since we injected mice spleens with HIV-infected PBMCs, our model was not designed to assess conventional homing of T cells to the brain, lymphoid tissues, and/or lymphoid associated tissues (e.g., GALT), and as expected, we did not observe anti-HIV duoCAR T cell localization to the brain, gut, and/or intestinal tissue of the mice. It is likely that T_SCM_/T_CM_ enriched anti-HIV duoCAR T cells will possess such homing capabilities as shown for a similar CAR T cell approach (54). While we cannot conclude that anti-HIV duoCAR T cells selectively home to lymphoid tissues, our study demonstrates that duoCAR T cells are capable of localizing to the site of HIV infection in the spleen, via circulating blood, resulting in potent in vivo HIV suppression and elimination of HIV^+^ cells. No apparent CAR-related toxicities, such as weight loss, behavioral changes, or premature animal death, were observed in mice treated with duoCAR T cells. Lack of CAR T cell penetration in the CNS may represent exclusion by the blood brain barrier and/or the absence of HIV infection in the brain, which takes place in the spleen of the hu-spl-PBMC-NSG mouse model system. While patients with cancer who were treated with CAR T cell products enriched for an early memory T cell phenotype demonstrated sustained remission, nonresponse was associated with treatment with CAR T cells predominantly of a late-memory phenotype (55, 56). T_SCM_ and T_CM_ enriched CAR T cell products are reported to overcome disease-related T cell defects such as T cell exhaustion (40, 42) and improve CAR T cell expansion and durability of the CAR T cell immune response (55, 56). Given the self-renewing properties of T_SCM_ cells, it is likely that T_SCM_/T_CM_-enriched duoCAR T cells will migrate to lymphoid tissues and display long-term persistence and efficacy, as shown for T_SCM_/T_CM_ enriched CD19 CAR T cells (40–44, 55, 56). Engraftment and longevity of T_SCM_/T_CM_ duoCAR T cells coupled with their broad killing capacity may enable long-term immune surveillance and sustained destruction of productive HIV-infected T cells and monocytes/macrophages residing in distal anatomical sites that harbor the HIV reservoir. Although expression of TCF-1, a transcription factor important for supporting an early memory phenotype and endowing T cells with proliferative capacity, was lower in duoCAR T cell products from PWH than products from HIV^–^ donors, the frequency of early memory–phenotypic cells is still substantially higher than is typically observed in other CAR T cell products during the ex vivo CAR T cell manufacturing process (57, 58). The observed T_SCM_/T_CM_ phenotype of anti-HIV duoCAR T cell products is likely reflective of the ex vivo CAR T cell manufacturing process, as reported for other CAR T cell therapies (40, 42, 43). It is plausible that the TCF-1^+^ duoCAR T cells would have an engraftment and proliferative advantage over unmodified cells, wherein these cells would expand in response to recrudescent HIV, giving rise to a greater population of TCF-1^+^ duoCAR T cells with self-renewal and memory capacity. The early memory phenotype combined with the markers of T cell activation and effector function of anti-HIV duoCAR T cell products suggest the CAR T cell manufacturing protocol generates a product that is both primed to attack cells with active HIV infection and that may provide a durable response over time in PWH. In vivo functional studies are under investigation in our clinical trial to corroborate the T_SCM_/T_CM_ phenotype of anti-HIV duoCAR T cells in PWH.

Monocytes/macrophages can be productively infected with HIV, albeit at lower levels than CD4^+^ T cells, and they contribute to the latent HIV reservoir in lymphoid tissues and the brain; they also constitute a significant barrier to an HIV cure (59). The increased resistance of HIV-infected monocytes/macrophages to HIV-induced cell death and immune-mediated clearance compared with HIV-infected T cells (48, 60) may be overcome by a CAR T cell approach (61). An important demonstration of our study is that anti-HIV duoCAR T cells are capable of effectively targeting and destroying HIV-infected monocytes, a HIV reservoir that is known to have lower gp120 antigen density on the surface of HIV-infected monocytes than HIV-infected CD4^+^ T cells (62).

In PWH, HIV infection can lead to immune dysregulation and functional impairment of HIV-specific T cells (i.e., CD8^+^ T cell exhaustion) even when viremia is controlled by ART (49). HIV-specific cytotoxic T lymphocytes (CTLs) play a critical role in controlling HIV infection (63, 64). Augmenting HIV-specific CTL immunity in PWH by providing help from CD4^+^ HIV-specific duoCAR T cells (6, 65) may enable durable control of HIV infection. An important finding of our study is the potent antiviral capacity of HIV-specific CD4^+^ and CD8^+^ duoCAR T cells that were derived from PWH donors on long-term suppressive ART. The anti-HIV duoCAR T cells from these donors potently suppressed cellular HIV infection from autologous immune cells superinfected with HIV. We have demonstrated that our manufacturing process coupled with the MND-ΔW duoCAR vector successfully reprogrammed and markedly expanded T cells derived from PWH donors at clinical scale without increasing the size of the HIV reservoir in the final anti-HIV duoCAR T cell product. Given that anti-HIV duoCAR T cells from these ART-suppressed individuals eliminated HIV-infected cells during acute and established in vitro infections, in vitro–based assays of HIV-dependent immune dysfunction may not mimic in vivo experiments in an otherwise immune-competent host.

One major advantage of using the MND-regulated duoCAR vector to generate anti-HIV duoCAR T cells is that it may be less prone to silencing in hematopoietic cell lineages due to the deletion of repressive cis-acting promoter elements (34, 35). Our study found that MND-regulated duoCAR T cells showed improved and durable duoCAR expression over a range of MOIs tested, concomitant with lower vector integration frequencies than the MSCV-regulated duoCAR vector. Although the inclusion of WPRE in the LV backbone was reported to improve LV titers and integrated transgene expression in human cells (66, 67), we observed that the WPRE was not required for optimal duoCAR expression in our study. Congruent with reports that promoter identity may augment the therapeutic efficacy of the CAR T cell product (37), MSCV- and MND-regulated anti-HIV duoCAR T cells displayed sustained anti-HIV activity, as evidenced by our demonstration of their ability to repeatedly eliminate HIV gp120–expressing cells in vitro (modeled using Env^+^ GFP^+^ target cells) and suppress productive HIV infection in vivo without signs of premature CAR T cell exhaustion or lack of persistence. Our previous studies have established the resistance profile of anti-HIV duoCAR T cells to HIV infection and the lack of off-target effects on human cells that do not express the HIV-1 Env glycoprotein (6). Durable anti-HIV responses displayed by anti-HIV duoCAR T cells will be crucial for limiting HIV replication and controlling viral loads in the body and, ultimately, for potentially achieving a functional cure for HIV.

Our current clinical trial (“CAR T cells for HIV infection”, NCT04648046) is designed to investigate anti-HIV duoCAR T cell therapy as a potential HIV remission strategy, using ART interruption to enable reactivation of latent reservoir cells and increased HIV-1 Env expression on their surface to render them susceptible to CAR-mediated elimination. CAR T cells may be limited in their ability to eliminate latent infected cells in the absence of reactivation.

Our results should be interpreted with the understanding that the hu-spl-PBMC-NSG mouse model used in our study has biological limitations, which is true for any animal model, including the BLT humanized mice model (68). Congruent with the BLT mouse model in its ability to evaluate CAR T cell efficacy (9), the hu-spl-PBMC-NSG model supports high viral loads supports CD4^+^ T cell depletion, and has the ability to rapidly assess anti-HIV CAR T cell efficacy, but it does so without the possible confounding effects of a reconstituted endogenous T cell immune response (6). While our current study does not demonstrate that duoCAR T cells containing anti-HIV Env–targeting binders can target and eliminate HIV-infected cells reactivated from latent HIV reservoirs, we have shown that the mD1.22 and m36.4 binders in the anti-HIV duoCAR T cells can target and recognize the HIV Env glycoprotein expressed on the surface of reactivated latent HIV-infected cell lines (69). While we found it challenging to reactivate HIV in T cells in vitro from PWH on long-term suppressive ART, anti-HIV duoCAR T cells will likely engender similar antiviral activities against HIV-infected cells reactivated from latency, as reported for other CAR T cell therapies targeting similar HIV-1 Env epitopes as those reported for our duoCAR (7, 15, 16, 24). Although traditional plasma viral load assays provide insight into HIV replication, quantification of cell-associated HIV infection by luciferase activity and cell-associated total HIV DNA directly measure CAR T cell–mediated suppression of productive HIV infection, resulting from viral replication, and capacity of CAR T cells to destroy HIV-infected cells. Further in-depth comparative analyses in vivo between anti-HIV duoCAR T cells derived from HIV seropositive and seronegative individuals would facilitate further understanding of the ability of redirected T cells from PWH to eliminate virus-infected cells that comprise the HIV reservoir. In conclusion, these IND-enabling studies support initiation of our presently open clinical trial (NCT04648046) to evaluate the safety and efficacy of anti-HIV duoCAR T cell therapy to treat HIV in ART-suppressed PWH.

## Methods

Supplemental Methods are available online with this article.

### Study design.

Sample sizes were determined based on our previous experience using the in vitro and in vivo models to generate statistically significant results. No samples or animals were excluded from the analysis. Blinding and randomization were not used during the study.

### Statistics.

Statistical analyses were conducted using 1-way or 2-way ANOVA with Tukey’s or Dunnett’s multiple-comparison post hoc tests (GraphPad Prism software, Version 8.4.3) except for Figure 4, A–C. Statistical analysis in Figure 4, A–C, was conducted via unpaired 2-tailed Student’s *t* test. A *P* value less than 0.05 was considered significant.

### Study approval.

All the mouse studies were approved by the IACUC and the IRB at Albert Einstein College of Medicine in compliance with the human and animal experimentation guidelines of the U.S. Department of Health and Human Services and in adherence to the *Guide for the Care and Use of Laboratory Animals* (National Academies Press, 2011).

## Author contributions

KAG, AR, and HS contributed equally to this work. KAG, AR, RLR, JWB, HG, and BD conceptualized and planned the study. KAG, AR, RLR, and HG wrote the original draft of the manuscript. KAG designed, constructed, and characterized the new anti-HIV duoCAR vectors. YW characterized the anti-HIV duoCAR vectors in HIV seronegative donors under the supervision of KAG. AR, HS, DL, VC, JW, and AB conducted the in vitro and in vivo HIV challenge/CAR T cell killing studies. CO and JCK provided the replication competent HIV-LucR IMC. SGD supervised recruitment of PWH in the study. RLR led CyTOF experiments performed by LZ and YYW. MDL and JSR provided oversight for anti-HIV duoCAR T cell manufacturing runs performed by SKB. DSD developed the anti-HIV binders (mD1.22 and m36.4) used to generate anti-HIV duoCAR T cells. KAG, HG, YX, SGD, DSD, CO, JCK, JWB, RO, and BD critically reviewed and edited the manuscript.

## Supplementary Material

Supplemental data

Supplemental table 1

## Figures and Tables

**Table 1:**
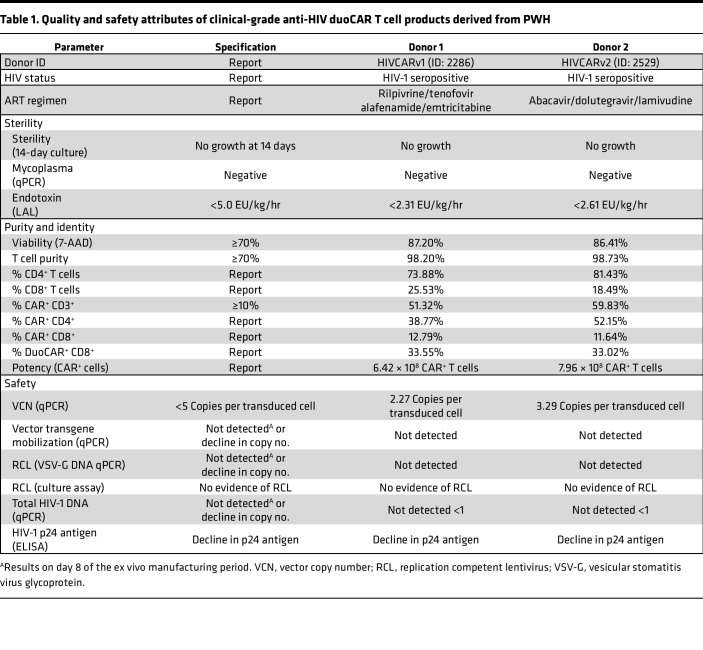
Quality and safety attributes of clinical-grade anti-HIV duoCAR T cell products derived from PWH

**Table 2:**
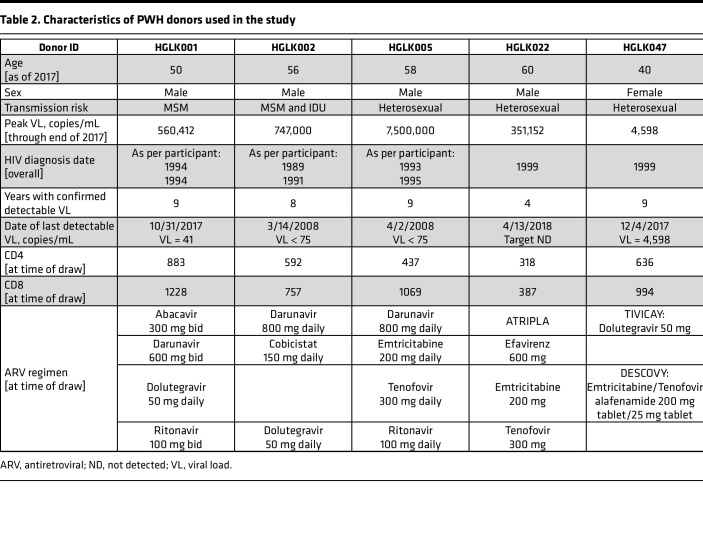
Characteristics of PWH donors used in the study

## Version 1 11/08/2022

Electronic publication

## References

[1] Schuster SJ (2019). Tisagenlecleucel in adult relapsed or refractory diffuse large B-cell lymphoma. N Engl J Med.

[2] Neelapu SS (2017). Axicabtagene ciloleucel CAR T-cell therapy in refractory large B-cell lymphoma. N Engl J Med.

[3] Locke FL (2019). Long-term safety and activity of axicabtagene ciloleucel in refractory large B-cell lymphoma (ZUMA-1): a single-arm, multicentre, phase 1-2 trial. Lancet Oncol.

[4] Abramson JS (2020). Lisocabtagene maraleucel for patients with relapsed or refractory large B-cell lymphomas (TRANSCEND NHL 001): a multicentre seamless design study. Lancet.

[5] Abramson JS (2019). Successful anti-CD19 CAR T-cell therapy in HIV-infected patients with refractory high-grade B-cell lymphoma. Cancer.

[6] Anthony-Gonda K (2019). Multispecific anti-HIV duoCAR T cells display broad in vitro antiviral activity and potent in vivo elimination of HIV-infected cells in a humanized mouse model. Sci Transl Med.

[7] Herzig E, Kim KC, Packard TA, Vardi N, Schwarzer R, Gramatica A (2019). Attacking Latent HIV with convertibleCAR-T Cells, a Highly Adaptable Killing Platform. Cell.

[8] Patasic L (2020). Designed ankyrin repeat protein (DARPin) to target chimeric antigen receptor (CAR)-redirected T cells towards CD4^+^ T cells to reduce the latent HIV^+^ cell reservoir. Med Microbiol Immunol.

[9] Maldini CR (2020). Dual CD4-based CAR T cells with distinct costimulatory domains mitigate HIV pathogenesis in vivo. Nat Med.

[10] Kuhlmann AS (2018). Chimeric antigen receptor T-cell approaches to HIV cure. Curr Opin HIV AIDS.

[11] Hale M (2017). Engineering HIV-resistant, anti-HIV chimeric antigen receptor T cells. Mol Ther.

[12] Haran KP (2018). Simian immunodeficiency virus (SIV)-specific chimeric antigen receptor-T cells engineered to target B cell follicles and suppress SIV replication. Front Immunol.

[13] Zhen A (2021). Robust CAR-T memory formation and function via hematopoietic stem cell delivery. PLoS Pathog.

[14] Zhen A (2017). Long-term persistence and function of hematopoietic stem cell-derived chimeric antigen receptor T cells in a nonhuman primate model of HIV/AIDS. PLoS Pathog.

[15] Liu B (2021). Broadly neutralizing antibody-derived CAR T cells reduce viral reservoir in HIV-1-infected individuals. J Clin Invest.

[16] Liu B (2016). Chimeric antigen receptor T cells guided by the single-chain Fv of a broadly neutralizing antibody specifically and effectively eradicate virus reactivated from latency in CD4+ T lymphocytes isolated from HIV-1-infected individuals receiving suppressive combined antiretroviral therapy. J Virol.

[17] Haeseleer F (2020). Real-time killing assays to assess the potency of a new anti-SIV CAR. AIDS Res Hum Retroviruses.

[18] Ariza-Heredia EJ (2017). False-positive HIV nucleic acid amplification testing during CAR T-cell therapy. Diagn Microbiol Infect Dis.

[19] Rust BJ (2020). Robust expansion of HIV CAR T cells following antigen boosting in ART-suppressed nonhuman primates. Blood.

[20] Hu D (2020). Improving safety of cancer immunotherapy via delivery technology. Biomaterials.

[21] Deeks SG (2002). A phase II randomized study of HIV-specific T-cell gene therapy in subjects with undetectable plasma viremia on combination antiretroviral therapy. Mol Ther.

[22] Mitsuyasu RT (2000). Prolonged survival and tissue trafficking following adoptive transfer of CD4zeta gene-modified autologous CD4(+) and CD8(+) T cells in human immunodeficiency virus-infected subjects. Blood.

[23] Scholler J (2012). Decade-long safety and function of retroviral-modified chimeric antigen receptor T cells. Sci Transl Med.

[24] Liu L (2015). Novel CD4-based bispecific chimeric antigen receptor designed for enhanced anti-HIV potency and absence of HIV entry receptor activity. J Virol.

[25] Lorenzo-Redondo R (2016). Persisten HIV-1 replication maintains the tissue reservoir during therapy. Nature.

[26] Neidleman J (2020). Phenotypic analysis of the unstimulated in vivo HIV CD4 T cell reservoir. Elife.

[27] Connick E (2007). CTL fail to accumulate at sites of HIV-1 replication in lymphoid tissue. J Immunol.

[28] Abreu CM (2019). Infectious virus persists in CD4^+^ T cells and macrophages in antiretroviral therapy-suppressed simian immunodeficiency virus-infected macaques. J Virol.

[29] Fukazawa Y (2015). B cell follicle sanctuary permits persistent productive simian immunodeficiency virus infection in elite controllers. Nat Med.

[30] Rainho JN (2015). Nef is dispensable for resistance of simian immunodeficiency virus-infected macrophages to CD8+ T cell killing. J Virol.

[31] Vojnov L (2012). The majority of freshly sorted simian immunodeficiency virus (SIV)-specific CD8(+) T cells cannot suppress viral replication in SIV-infected macrophages. J Virol.

[32] Igarashi T (2001). Macrophage are the principal reservoir and sustain high virus loads in rhesus macaques after the depletion of CD4+ T cells by a highly pathogenic simian immunodeficiency virus/HIV type 1 chimera (SHIV): implications for HIV-1 infections of humans. Proc Natl Acad Sci U S A.

[33] Halene S (1999). Improved expression in hematopoietic and lymphoid cells in mice after transplantation of bone marrow transduced with a modified retroviral vector. Blood.

[34] Astrakhan A (2012). Ubiquitous high-level gene expression in hematopoietic lineages provides effective lentiviral gene therapy of murine Wiskott-Aldrich syndrome. Blood.

[35] Cartier N (2009). Hematopoietic stem cell gene therapy with a lentiviral vector in X-linked adrenoleukodystrophy. Science.

[36] Edmonds TG (2010). Replication competent molecular clones of HIV-1 expressing Renilla luciferase facilitate the analysis of antibody inhibition in PBMC. Virology.

[37] Eyquem J (2017). Targeting a CAR to the TRAC locus with CRISPR/Cas9 enhances tumour rejection. Nature.

[38] Lugli E (2013). Superior T memory stem cell persistence supports long-lived T cell memory. J Clin Invest.

[39] Lugli E (2013). Identification, isolation and in vitro expansion of human and nonhuman primate T stem cell memory cells. Nat Protoc.

[40] Arcangeli S (2020). Next-generation manufacturing protocols enriching T_SCM_ CAR T cells can overcome disease-specific T cell defects in cancer patients. Front Immunol.

[41] Xu Y (2014). Closely related T-memory stem cells correlate with in vivo expansion of CAR.CD19-T cells and are preserved by IL-7 and IL-15. Blood.

[42] Jackson Z (2020). Automated manufacture of autologous CD19 CAR T cells for treatment of non-hodgkin lymphoma. Front Immunol.

[43] Blaeschke F (2018). Induction of a central memory and stem cell memory phenotype in functionally active CD4^+^ and CD8^+^ CAR T cells produced in an automated good manufacturing practice system for the treatment of CD19^+^ acute lymphoblastic leukemia. Cancer Immunol Immunother.

[44] Ghassemi S (2018). Reducing ex vivo culture improves the antileukemic activity of chimeric antigen receptor (CAR) T cells. Cancer Immunol Res.

[45] Paugh BS (2021). Reference standards for accurate validation and optimization of assays that determine integrated lentiviral vector copy number in transduced cells. Sci Rep.

[46] Kratchmarov R (2018). TCF1 expression marks self-renewing human CD8^+^ T cells. Blood Adv.

[47] Rutishauser RL (2021). TCF-1 regulates HIV-specific CD8+ T cell expansion capacity. JCI Insight.

[48] Clayton KL (2018). Resistance of HIV-infected macrophages to CD8^+^ T lymphocyte-mediated killing drives activation of the immune system. Nat Immunol.

[49] Perdomo-Celis F (2019). An altered cytotoxic program of CD8+ T-cells in HIV-infected patients despite HAART-induced viral suppression. PLoS One.

[50] Moschovakis GL (2019). The chemokine receptor CCR7 is a promising target for rheumatoid arthritis therapy. Cell Mol Immunol.

[51] Chauveau A (2020). Visualization of T cell migration in the spleen reveals a network of perivascular pathways that guide entry into T zones. Immunity.

[52] Lee SH (2020). Feasibility of real-time in vivo 89Zr-DFO-labeled CAR T-cell trafficking using PET imaging. PLoS One.

[53] Pittet MJ (2007). In vivo imaging of T cell delivery to tumors after adoptive transfer therapy. Proc Natl Acad Sci U S A.

[54] Barber-Axthelm IM (2021). Stem cell-derived CAR T cells traffic to HIV reservoirs in macaques. JCI Insight.

[55] Berdeja JG (2019). Updated results from an ongoing phase 1 clinical study of bb21217 anti-Bcma CAR T cell therapy. Blood.

[56] Fraietta JA (2018). Determinants of response and resistance to CD19 chimeric antigen receptor (CAR) T cell therapy of chronic lymphocytic leukemia. Nat Med.

[57] Hollyman D (2009). Manufacturing validation of biologically functional T cells targeted to CD19 antigen for autologous adoptive cell therapy. J Immunother.

[58] Sommermeyer D (2016). Chimeric antigen receptor-modified T cells derived from defined CD8+ and CD4+ subsets confer superior antitumor reactivity in vivo. Leukemia.

[59] Wong ME (2019). The HIV reservoir in monocytes and macrophages. Front Immunol.

[60] Vojnov L (2012). The majority of freshly sorted simian immunodeficiency virus (SIV)-specific CD8(+) T cells cannot suppress viral replication in SIV-infected macrophages. J Virol.

[61] Clayton KL (2021). HIV-infected macrophages resist efficient NK cell-mediated killing while preserving inflammatory cytokine responses. Cell Host Microbe.

[62] Crowe SM (1992). Human immunodeficiency virus-infected monocyte-derived macrophages express surface gp120 and fuse with CD4 lymphoid cells in vitro: a possible mechanism of T lymphocyte depletion in vivo. Clin Immunol Immunopathol.

[63] Deng K (2015). Broad CTL response is required to clear latent HIV-1 due to dominance of escape mutations. Nature.

[64] Fan J (2019). CTL-mediated immunotherapy can suppress SHIV rebound in ART-free macaques. Nat Commun.

[65] Maldini CR (2020). HIV-resistant and HIV-specific CAR-modified CD4^+^ T cells mitigate HIV disease progression and confer CD4^+^ T cell help in vivo. Mol Ther.

[66] Schambach A (2006). Woodchuck hepatitis virus post-transcriptional regulatory element deleted from X protein and promoter sequences enhances retroviral vector titer and expression. Gene Ther.

[67] Zufferey R (1999). Woodchuck hepatitis virus posttranscriptional regulatory element enhances expression of transgenes delivered by retroviral vectors. J Virol.

[68] Karpel ME (2015). BLT humanized mice as a small animal model of HIV infection. Curr Opin Virol.

[69] Bardhi A (2017). Potent in vivo NK cell-mediated elimination of HIV-1-infected cells mobilized by a gp120-bispecific and hexavalent broadly neutralizing fusion protein. J Virol.

