# Supplementary material for: In vivo killing of primary HIV-infected cells by peripheral-injected early memory–enriched anti-HIV duoCAR T cells
Source: JCI Insight. 2022 Nov 8;7(21):e161698. doi: 10.1172/jci.insight.161698 (PMC9675454; doi:10.1172/jci.insight.161698)
Supplement: Supplemental data [file jciinsight-7-161698-s172.pdf]

**Supplemental material**

## Methods

### Construction of anti-HIV duoCAR transfer plasmids

The MSCV-ΔW duoCAR transfer plasmid was constructed by excising the WPRE sequence from the MSCV+W (D13) transfer plasmid using Bsi WI and Swa I restriction enzyme sites. The Bsi WI 5' overhang was filled in using the DNA polymerase Large (Klenow) Fragment as per the manufacturer's instructions (New England Biolabs) followed by blunt-end self-ligation. The ligation reaction was transformed into chemical-competent *E. coli* cells (Stellar chemical-competent cells, Takara), plated overnight on Kanamycin LB plates, and clones were screened by Sanger sequencing. The MND-ΔW duoCAR plasmid was generated by replacing the MSCV promoter with the MND promoter using Hpa I and Xho I cloning sites followed by cohesive-end cloning using the Quick Ligation T4 DNA Ligase kit (New England Biolabs) as per the manufacturer's instructions.

### Generation of anti-HIV duoCAR lentiviral vectors and titer determination

Anti-HIV duoCAR lentiviral vectors were generated at research-scale using a previously described method (6) and transducing titers were determined by qPCR. Briefly, on day 0, 3 x $10^5$ SupT1 cells were transduced with 100 µl of a LV dilution series and 24 hours later, the transduced cells were fed with 1 mL of complete RPMI media (RPMI media supplemented with 10% fetal bovine serum). On day 3, transduced SupT1 cells were collected, washed with sterile 1x PBS (phosphate-buffered saline, pH 7.4), and genomic DNA was extracted using the Qiagen DNeasy blood and tissue extraction kit (Qiagen). Eluted genomic DNA was subjected to qPCR using a proprietary set of primers and probe specific to the truncated gag gene in the LV backbone and normalized to the PTBP2 gene. Transducing titer was calculated based on vector copy number and expressed as transducing units per milliliter (TU/mL).

**Generation of anti-HIV duoCAR-T cell products on the CliniMACS Prodigy**

For clinical-scale CAR-T cell manufacturing, leukopaks were obtained from Key Biologics ($HIV^-$) or Vitalant ($HIV^+$) and shipped overnight priority to Lentigen Technology, Inc. (LTI) or the Cellular Therapy Lab of University Hospitals Cleveland Medical Center Seidman Cancer Center/Case Western Reserve University (UH/CWRU) Center for Regenerative Medicine, respectively. Upon arrival, the leukopak was allowed to reach room temperature and up to 60 mL of the leukopak was aseptically transferred to a sterile transfer bag and subsequently diluted to 100 mL with CliniMACS PBS-EDTA buffer before loading on the CliniMACS Prodigy® device. The Prodigy device was equipped with a TS520 tubing set and GMP-grade reagents were loaded onto the device as prompted by the device's graphical user interface (GUI). All steps were performed in the TS520 tubing set and its cultivation chamber (e.g., enrichment, activation, transduction, and expansion) unless otherwise indicated. On day 0, $CD4^+$ and $CD8^+$ T cells were enriched via positive selection using GMP-grade CD4 and CD8 microbeads (Miltenyi Biotec) and subsequently activated with anti-CD3/anti-CD28 TransAct reagent (Miltenyi Biotec) in TexMACS media supplemented with recombinant human IL2 (200 U/mL). The following day (day 1) activated $CD4^+$ and $CD8^+$ T cells were transduced with the anti-HIV duoCAR vector (MND-ΔW duoCAR) at a MOI of 20. The anti-HIV duoCAR vector was removed on day 3 of the culture via a series of wash steps. Transduced T cells were fed every one or two days via media exchanges with TexMACS medium supplemented with IL2 (200 U/mL). On day 8, the cells were harvested on the Prodigy device in a sterile collection bag and formulated in PlasmaLyte A. The duoCAR-T cells were removed from the Prodigy device followed by transfer to a sterilized biosafety cabinet for further formulation and cryopreservation. Products manufactured in the Cellular Therapy Laboratory at CWRU were subjected to rigorous release testing to demonstrate quality and safety of the duoCAR-T cells products as per institutional standard operating procedures. Products were evaluated for viability (7-AAD), T cell purity, CAR identity, sterility (14-day culture), Mycoplasma

(QPCR), and Endotoxin (LAL) testing by the Cellular Therapy Laboratory at CWRU using institutional standard operating procedures. The duoCAR-T cell products were cryopreserved in PlasmaLyte A containing DMSO and stored long-term at < -150°C in the vapor phase of liquid nitrogen.

**Generation of anti-HIV duoCAR-T cells from HIV seronegative and seropositive donors**

PBMCs from HIV seronegative and HIV seropositive donors were collected by leukapheresis using a protocol approved by the Albert Einstein College of Medicine Institutional Review Board, isolated by ficoll-paque gradient separation, aliquoted, frozen, and stored at -130°C. Alternatively, PBMCs from leukapheresed HIV seronegative donors were purchased from Key Biologics and processed by Lentigen Technology, Inc. according to standard operating procedures. Anti-HIV duoCAR-T cells were generated from HIV seronegative donors as previously described (6). For HIV seropositive donors, $CD4^+$ and $CD8^+$ T cells were isolated from fresh-thawed frozen PBMCs by immunomagnetic sorting using CD4 and CD8 magnetic beads (Miltenyi Biotec) were activated by incubation with T cell TransAct reagent (Miltenyi Biotec) for one day in TexMACS GMP-grade media (Miltenyi Biotec) supplemented with recombinant human IL2 (100 U/mL), penicillin (100 U/mL) and streptomycin (10 µg/mL). After washing, the activated T cells were transduced with MSCV+W, MSCV-ΔW, or MND-ΔW lentiviral vector at a MOI of 20. After two days of culture, the transduced cells were extensively washed with PBS, and cultured and expanded for an additional seven days. For research scale productions, Darunavir (0.25 □M) was added to all cultures of PBMCs from HIV-seropositive donors. Lentiviral vector transduction of primary T cells was confirmed by flow cytometric analysis. Cells were stained using VioBlue®-conjugated anti-CD3 (1:100 dilution, Miltenyi Biotec, Catalog No. 130-113-133/Clone BW264/56), FITC-conjugated anti-CD8 (1:100 dilution, Miltenyi Biotec, Catalog No. 130-113-157/Clone BW135/80), PE-conjugated anti-CD4 (1:50 dilution, Miltenyi Biotec, Catalog No. 130-113-214/Clone VIT4), and anti-m36.4 idiotype antibody (1:1000, Lentigen Technology, Inc., Clone: 51) plus Alexa Fluor 647-

conjugated goat anti-human Fc IgG (1:200, Southern Biotech, Catalog No. 2048-31/Clone: N/A) as previously described (6) and analyzed in Supplemental Figure 1.

**Flow cytometry detection of anti-HIV duoCAR-T cells**

Briefly, 3 x $10^5$ anti-HIV duoCAR-T cells or untransduced T cells were first stained with Fc-conjugated anti-m36.4 idiotype antibody (1 mg/mL, Lentigen Technology, Inc., Clone: 51.) in wash buffer (1x PBS, pH 7.4 supplemented with 0.5% BSA) for 30 minutes at 4°C. The cells were washed twice to remove unbound antibody via centrifugation (1,000 rpm) for 5 minutes at 4°C. The cells were stained with a cell viability dye (7-AAD, 1:10; Miltenyi Biotec, Catalog No. 130-111-568/Clone: N/A) and a cocktail of antibodies purchased from Miltenyi Biotec, unless otherwise indicated, containing PE-conjugated anti-CD3 (1:100, Miltenyi Biotec, Catalog No. 130-113-129/Clone: BW264/56), VioBlue®-conjugated anti-CD4/mD1.22 (1:100, Miltenyi Biotec, Catalog No. 130-113-219/Clone: VIT4), PE-Vio770-conjugated anti-CD8 (1:100, Miltenyi Biotec, Catalog No. 130-113-159/Clone: BW135/80), and AlexaFluor647-conjugated goat anti-human Fc IgG (1:200, Southern Biotech, Catalog No. 2048-31/Clone: N/A) for 30 minutes at 4°C and then were washed twice as described above. Stained cells were resuspended in wash buffer (300 µL) and analyzed using the MQ10 flow cytometry analyzer (Miltenyi Biotec). Prior to start of the analysis, the instrument was properly calibrated using flow cytometry calibration beads (Miltenyi Biotec) and compensated using single stain controls. For T cell memory phenotyping, the pre-infusion (Day 8, harvest) anti-HIV duoCAR-T cell product was stained with an antibody cocktail (T cell memory panel) consisting of APC-Vio770-conjugated anti-CD45RA (Miltenyi Biotec, Catalog No. 130-113-915/Clone: T6D11), PE-Vio770-conjugated anti-CCR7 (Miltenyi Biotec, Catalog No. 130-118-488/Clone: REA108) and FITC-conjugated anti-CD95 (Miltenyi Biotec, Catalog No. 130-124-214/Clone: DX2). Within the live lymphocyte gate (>95% $CD3^+$ T cells), the T cell memory markers CD45RA and CCR7 were used to stratify T cell populations into naive ($T_N$), central memory ($T_{CM}$), effector memory ($T_{EM}$), and effector memory T cells re-expressing

CD45RA ($T_{EMRA}$). Early-memory stem cell or stem-cell like ($T_{SCM}$) cells were differentiated from $T_N$ cells based on co-expression of CCR7 and CD95. Flow cytometry gates were set using the appropriate biological (e.g., UTD T cell control) and/or fluorescence minus one (FMO) control prior to start of the run. At minimum, 30,000 events were acquired for each sample. FlowJo version 10 software was used to analyze cell populations.

**Long-term co-culture cytotoxicity assay using HEK 293T-Env$^+$ GFP$^+$ target cells**

The long-term killing efficacy of anti-HIV duoCAR-T cells was evaluated by challenging anti-HIV duoCAR-T cells with HEK293T-Env$^+$Luc$^+$GFP$^+$ (Env$^+$GFP$^+$) target cells on a weekly basis over a 17-day period. Briefly, on day 0, the anti-HIV duoCAR-T cells ($0.6 \times 10^6$ cells/well) were co-cultured with Env$^+$GFP$^+$ target cells ($2 \times 10^6$ cells/well) at an effector to target (E:T) ratio of 0.3:1 in TexMACS medium without cytokine supplementation. To determine CAR-mediated killing of Env$^+$GFP$^+$ target cells, a portion of the co-cultured cells was evaluated by flow cytometry at baseline (day 0) and on days 1, 4, and 7 post-challenge via detection of residual target cell numbers using Absolute Counting Beads (Thermo Fisher Scientific). The co-cultured cells were stained with the 7-AAD cell viability dye to determine cytotoxicity-induced target cell apoptosis in the Env$^+$GFP$^+$ cell population. Flow cytometry data was acquired on the MQ10 flow cytometer (Miltenyi Biotec) and analyzed by Flow Jo version 10 software (Tree Star). At the end of the first round of co-culture, cells in the culture were counted and fresh Env$^+$GFP$^+$ target cells were added at an approximate E:T ratio of 0.3:1. The second and third challenge round were conducted in the same way as the first round.

**Infectious molecular clones of HIV-1 *Renilla* luciferase (LucR) reporter viruses**

We utilized HIV infectious molecular clones (IMC) which encode the *Renilla* luciferase (LucR) reporter as a surrogate marker to quantify productive HIV infection. As previously described (67), HIV-LucR-IMC were generated by engineering the replication competent subtype B HIV IMC,

pNL4-3, to express, in *cis*, the LucR reporter gene and heterologous *env* gene sequences of selected HIV-1 strains. Virus stocks were produced by transfection of 293T cells with the indicated proviral plasmid DNAs, and the infectious titers (IU/mL) were determined on TZM-bl cells as previously described (36). HIV-LucR IMC with *env* sequences from clades A, B, and C HIV-1 strains used in this study were NL-LucR.T2A-A.396-F1_F6_20.ecto (clade A), NL-LucR.T2A-BaL.ecto (clade B; BaL *env* accession number AY426110), NL-LucR.T2A-B.CH077.ecto (clade B), and NL-LucR.T2A-C.Du151.2.ecto (clade C) (36, 67, 68).

**In vitro CAR-T cell mediated killing of HIV-infected PBMCs assay**

The capacity of duoCAR-T cells derived from seronegative donors to eliminate acute HIV-infected cells in vitro was quantified as we previously described (6). For studies using PWH donors, $CD8^+$ T cells were depleted from donor PBMCs by incubating with anti-CD8 magnetic beads (Miltenyi Biotec) followed by T cell activation with phytohemagglutinin A (PHA, 4 µg/mL). Activated T cells were cultured for 2 days in complete RPMI media supplemented with 10% fetal bovine serum and recombinant human IL2 (100 IU/mL), washed, plated in a 96-well U bottom plate (100,000 cells/well) and spinfected with the indicated HIV-LucR IMC virus (~$1x10^6$ IU/mL). Autologous duoCAR T cells or untransduced (UTD) control T cells were added at an effector to target (E:T) ratio of 1:1 and cultured for 3 days (acute infection). Alternatively, spinfected cells were cultured for 3 days to establish HIV infection followed by addition of syngeneic CAR T cells or UTD control T cells at an effector to target (E:T) ratio of 1:1 and cultured for an additional 3 days. The cells were then lysed, and the magnitude of HIV infection was quantified by measuring LucR levels using the Renilla Luciferase System (Promega) as previously described (36, 67).

**In vitro CAR-mediated killing of HIV-infected monocytes assay**

Monocytes were isolated and cultured as previously described (69). Briefly, uninfected PBMCs from leukopaks obtained from the New York Blood Center were isolated by Ficoll density gradient

centrifugation using Ficoll-Paque Plus (GE Healthcare). $CD14^+$ monocytes were isolated by positive selection from PBMCs using anti-CD14 magnetic microbeads (Miltenyi Biotec). The isolated monocytes were cultured (2 x $10^6$ cells/mL) for 3 days non-adherently in Teflon flasks with complete RPMI media containing macrophage colony-stimulating factor (M-CSF, 10 ng/mL) (Peprotech) to increase the mature $CD14^+CD16^+$ monocyte subpopulation (70, 71). After 3 days, monocytes (10 x $10^6$ cells/mL) were either uninfected or infected with HIV BaL-LucR virus (~1x$10^6$ IU/mL). After 8 hours, the cells were centrifuged, and the excess virus was removed, resuspended in fresh media with M-CSF (2 x $10^6$ cells/mL), and cultured non-adherently in Teflon flasks for additional 2 days to facilitate HIV infection and replication. In parallel, PHA-stimulated PBMCs from the same donor were spinfected with HIV-BaL-LucR (~1x$10^6$ IU/mL) and cultured for 3 days in complete R10 media supplemented with recombinant human IL2 (100 U/mL). The infected monocytes or PBMCs (1 x $10^5$ cells/well) were either untreated or treated with autologous untransduced T cells or MND-ΔW duoCAR-T cells (1 x $10^5$ cells/well) at an E:T ratio of 1:1 for another 3 days. The cells were then lysed, and the magnitude of HIV infection was quantified by measuring LucR levels using the Renilla Luciferase System (Promega) as previously described (36, 67).

**Detection of total cell-associated HIV DNA in mice splenocytes using real-time qPCR**

Cell-associated total HIV DNA qPCR was performed by BioQual, Inc. as previously described (6). Briefly, genomic DNA was isolated from one million total $CD4^+$ and $CD8^+$ T cells at baseline and at various time points during the CAR-T cell manufacturing process using the DNeasy Blood and Tissue kit (Qiagen) as per the manufacturer’s instruction. Eluted DNA was subjected to real-time qPCR using primers and probes specific for wild type Gag HIV-1 and normalized to human β-actin gene. Total HIV DNA results were expressed as copies of total HIV DNA per $10^6$ β-actin equivalents.

**Vector copy number (VCN) assay**

VCN was determined for anti-HIV duoCAR-T cell products using the cFrag qPCR assay as previously described by our group (6). Results were expressed as average vector copy number per cell, per transduced cell, or per $10^6$ PTBP2 copies as indicated in the study.

**HIV p24 antigen ELISA**

The concentration of HIV p24 antigen in supernatants from cultured CAR-T cells from PWH was quantified using the HIV-1 p24 Antigen Capture Assay (ABL, Inc.) according to the manufacturer's instructions.

**Replication competent lentivirus (RCL) culture assay**

The RCL culture assay including the RCL (VSV-G DNA) QPCR assay was conducted by the Indiana University Gene Therapy Testing Laboratory under the direction of Dr. Kenneth Cornetta or Case Western Reserve University (RCL QPCR) as per standard operating procedures.

**Intravenous administration of duoCAR-T cell therapy in the hu-spl-PBMC-NSG mouse model of HIV infection**

We evaluated the in vivo capacity of intravenously administered CAR-T cells to suppress HIV infection using a modified version of the hu-spl-PBMC-NSG mouse model we previously described (6, 67). Three days after HIV-seronegative donor PBMCs were activated with PHA-L (4 □g/mL) and cultured in complete R10 media with added recombinant human IL2 (100 U/mL), the cells were washed and spinfected with C.Du151.2-HIV-LucR-IMC (~1 x $10^6$ IU/1 x $10^7$ activated PBMCs) for 90 minutes at 2,500 rpm (1,139 *x g*). Cells were placed on ice until time of injection and spinfected PBMCs (~1 x $10^7$) were intrasplenically injected into 7-10-week-old male NOD/SCID/IL2Rgamma$^{-/-}$ (NSG) mice (The Jackson Laboratory). Autologous anti-HIV duoCAR-T cells or UTD control T cells (2 x $10^6$ total cells to provide an E:T ratio of 1: 5) were intravenously

injected into the tail vein and the mice were monitored for the duration of the study for behavioral changes, overt phenotypic abnormalities, and weight loss as a potential consequence of the injected CAR-T cells. Mice were euthanized after 17-18 days and the spleens from one group of mice were harvested, homogenized into single cells suspensions, red blood cells were lysed, and total splenocytes were partitioned to analyze luciferase levels, $CD4^+$ and $CD8^+$ T cell populations, total cell-associated (CA)-HIV DNA, and the CAR-T cell population. Splenocyte luciferase levels were measured using the *Renilla* Luciferase Assay System (Promega) as previously described (6, 67).

**Flow cytometric analysis of $CD4^+$ and $CD8^+$ T cell populations in the mouse spleens**

After the mouse spleens were harvested, they were dissociated to generate a single-cell suspension which was treated with red cell lysis buffer to remove red blood cells, pelleted, and washed three times in MACS wash buffer (Miltenyi Biotec). To detect $CD4^+$ and $CD8^+$ T cell populations, cells were stained for 30 minutes on ice with APC-Cy7-conjugated anti-human CD45 (1:100 dilution, Cell Signaling Technology, Catalog No. 77322/Clone), VioBlue®-conjugated anti-CD3 (1:100 dilution, Miltenyi Biotec, Catalog No. 130-113-133/Clone BW264/56), FITC-conjugated anti-CD8 (1:100 dilution, Miltenyi Biotec, Catalog No. 130-113-157/Clone BW135/80), and PE-conjugated anti-CD4 (1:50 dilution, Miltenyi Biotec, Catalog No. 130-113-214/Clone VIT4). Cells were then washed twice with MACS buffer and fixed in 2% paraformaldehyde for 20 minutes on ice. Flow cytometry was performed on a BD LSR II flow cytometer (BD Biosciences) and analysis was performed using FlowJo software (Tree Star).

**Determination of anti-HIV duoCAR-T cell persistence and biodistribution in mice**

Blood and major organs (brain, lungs, liver, heart, blood, spleen, and intestinal/gut) were harvested from humanized NSG mice intravenously injected with CAR-T cells (2 x $10^6$ cells) and flash frozen for the detection of CAR-specific DNA in blood and tissues. CAR-T biodistribution

and persistence were quantified by the cFrag qPCR assay as we previously described (6). Results in the study were expressed as cFrag copies per million PTBP2 copies.

**Mass cytometry by Time-Of-Flight (CyTOF) analysis**

Cryopreserved untransduced (d0) and duoCAR-T cell (d8) enriched $CD4^+$ and $CD8^+$ T cells from uninfected and PWH donors were thawed, and 1-2 million cells were stained for mass cytometry following a previously published protocol (72, 73). A list of CYTOF antibodies is provided in Supplemental data file S1. Briefly, dead cells were removed by incubating the samples for one minute with 25mM Cisplatin (Sigma-Aldrich) in phosphate buffered saline (PBS) containing EDTA. Surface staining was performed using metal-tagged antibodies in PBS with 0.5% bovine serum albumin (BSA) for 30 minutes at room temperature followed by fixation and cells were permeabilized following manufacturer's instructions for the eBioscience Foxp3/Transcription Factor Staining Buffer Set (Thermo Fisher Scientific). Samples were barcoded using mass-tag cellular barcoding reagents diluted in Maxpar Barcode Perm Buffer (Fluidigm) as described previously (73). We combined up to twenty barcoded samples into a single tube and performed intracellular staining with antibodies diluted in eBioscience Foxp3/Transcription Factor kit perm wash (Thermo Fisher Scientific). Cells were fixed in freshly prepared 2% paraformaldehyde (Electron Microscopy Sciences, Hatfield, PA, USA) in the presence of a DNA intercalator (74), and then washed and analyzed on the Fluidigm CyTOF 2 mass cytometer within one week of staining.

**Data quality control for CyTOF analysis**

Following CyTOF data acquisition, the FCS files were normalized using bead standards and the data normalization algorithm using the R package 'premessa' (https://github.com/ParkerICI/premessa). The live cell events were debarcoded using a single-cell debarcoding algorithm (75) and we analyzed >25,000 (mostly >50,000) cells per sample.

Normalization beads were excluded based on Ce140 and Eu153 signal, single cell events were identified based on Ir191 DNA signal measured against event length, and $CD45^-$ or $Pt195^+$ dead cells were excluded. Potential batch effects were minimized by including samples from the same individual in the same experiment. Gating and heatmap generation were performed using CellEngine (CellCarta, Montreal, Canada).

# Supplemental Figure 1

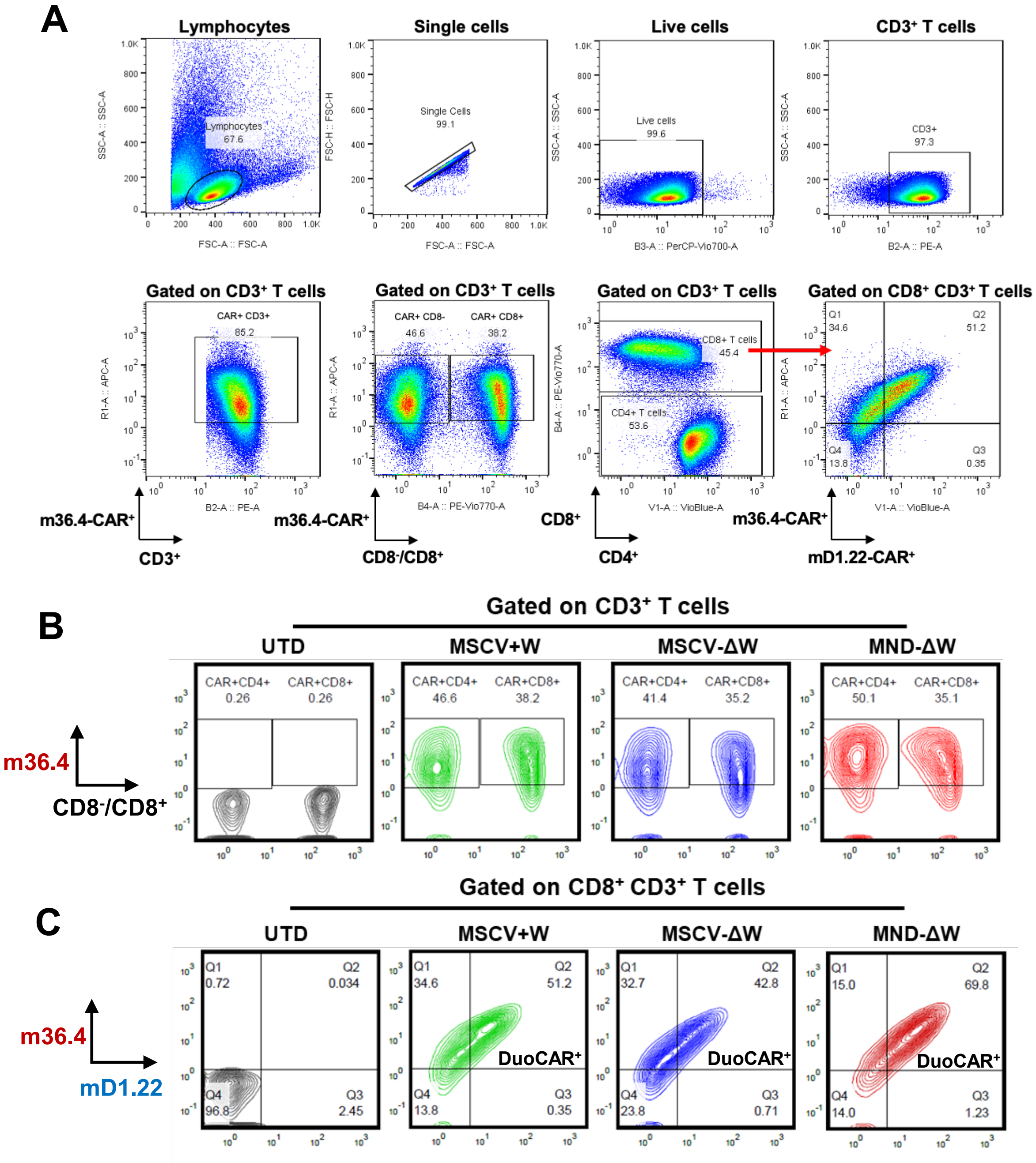


**Supplemental Figure 1. Gating strategy for vector-optimized anti-HIV duoCAR-T cell detection.** (**A**) Anti-HIV duoCAR-T cells (shown) were stained with an antibody cocktail consisting of PE-conjugated anti-CD3, VioBlue-conjugated anti-CD4, PE-Vio770-conjugated anti-CD8, and the anti-m36.4 CAR-specific detection reagent (APC channel) as described in Materials and Methods. Untransduced (UTD) control T cells (not shown) were used as a biological control to set gates for CAR positivity. The stained cells were gated on lymphocytes based on forward (FSC-A) and side scatter (SSC-A) followed by exclusion of cell doublets from the analysis (FSC-A versus FSC-H). Live cells (7-AAD negative) were discriminated from dead cells (7-AAD positive) via 7-AAD staining. Within the live $CD3^+$ T cell population, $CAR^+$ (m36.4-$CAR^+$) total T cells, T cell subsets ($CD4^+$ (CD8-) and $CD8^+$) and $duoCAR^+$ (mD1.22- and m36.4-$CAR^+$) $CD8^+$ T cells were identified. A shift in CD4 (mD1.22-CAR) and m36.4 mean fluorescence intensity (MFI) relative to UTD control T cells (not shown in the analysis) on $CD8^+$ T cells was used to determine % $duoCAR^+$ $CD8^+$ T cells (Q2 of the flow plot diagram) for anti-HIV duoCAR-T cell products. (**B**,**C**) Representative detection of total CAR expression on the surface of LV-modified (**B**) $CD4^+$ ($CD8^-$ gate) and $CD8^+$ T cells via anti-m36.4 flow cytometry and (**C**) duoCAR expression on the surface of $CD8^+$ T cells via anti-CD4 (mD1.22) and anti-m36.4 flow cytometry (Q2 of the flow plot shows $duoCAR^+$ $CD8^+$ T cells).

# Supplemental Figure 2

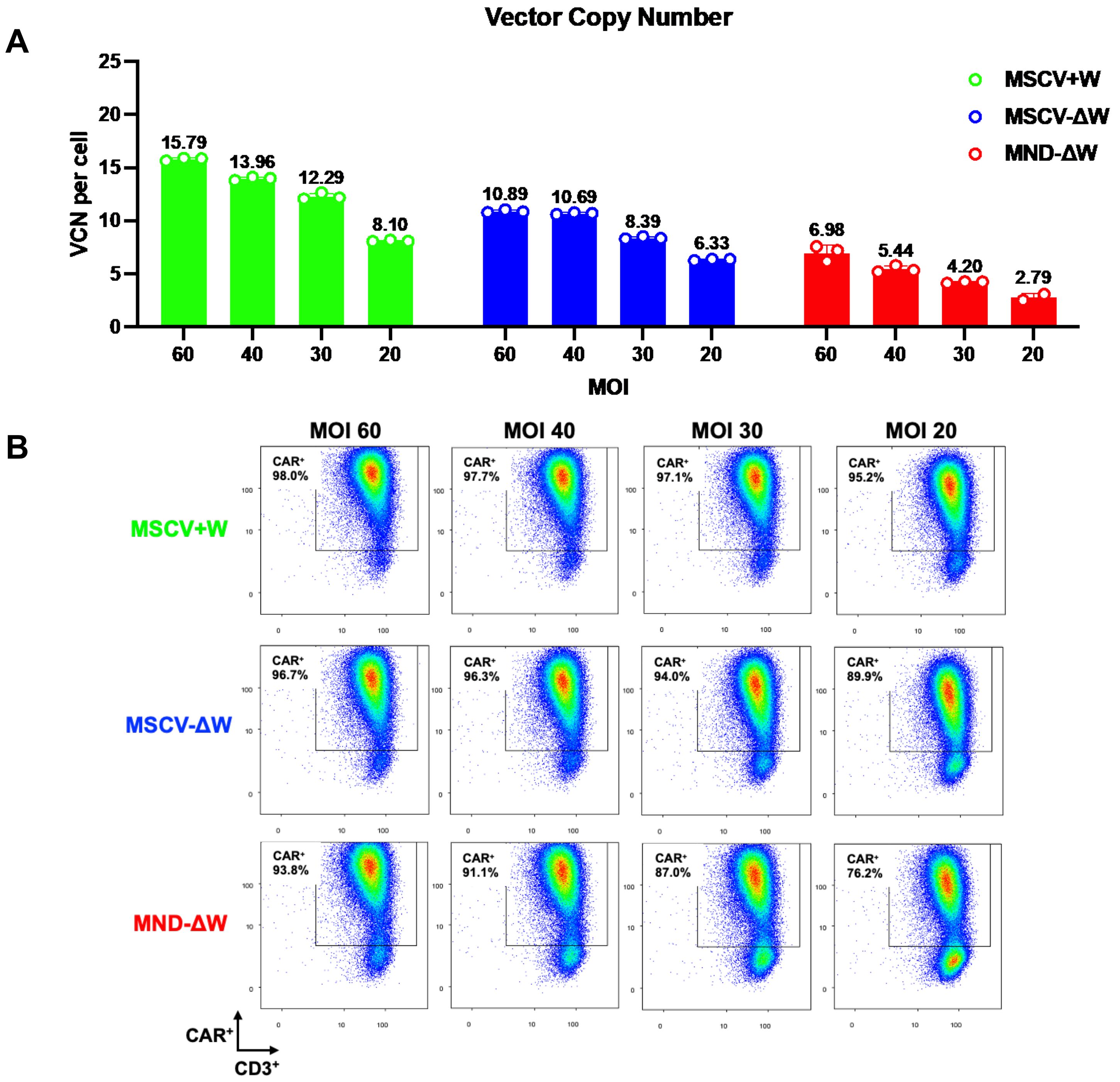


**Supplemental Figure 2. Determination of the optimal MOI for anti-HIV duoCAR vector transduction in primary T cells.** (**A**) Determination of vector copy number (VCN) as a function of multiplicity of infection (MOI) in primary T cells transduced with anti-HIV duoCAR LV constructs. (**B**) Determination of CAR transduction efficiency as a function of MOI in human primary T cells transduced with anti-HIV duoCAR LV constructs. Detection of the m36.4-CAR (CAR$^+$) in CD3$^+$ T cells serves as a LV transduction marker and was used to determine duoCAR transduction efficiency in LV-modified anti-HIV duoCAR-T cells as described in Materials and Methods.

# Supplemental Figure 3

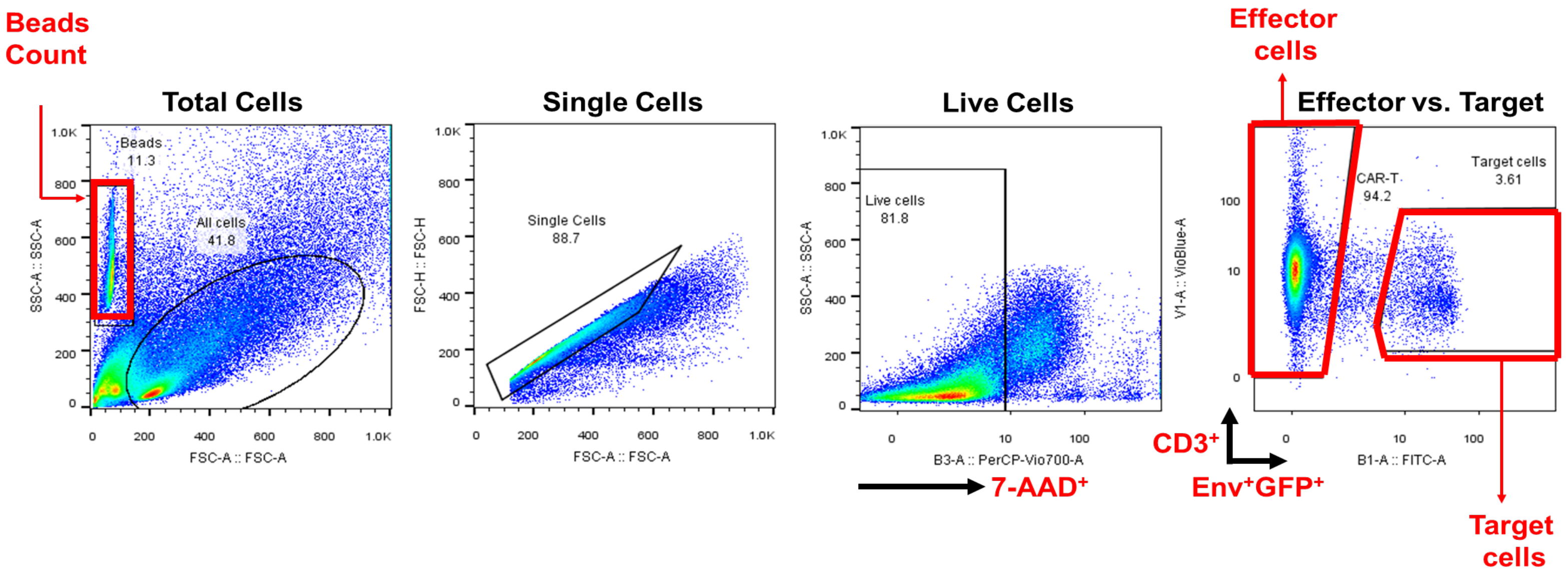


**Supplemental Figure 3. Gating strategy for long-term cytotoxicity assay.** Anti-HIV duoCAR-T cells were challenged with HEK293T-engineered $Env^{+}GFP^{+}$ cells and stained with VioBlue-conjugated anti-CD3 to discriminate total effector T cells ($CD3^{+}$ $GFP^{-}$ cells) from $GFP^{+}$ Env-expressing target cells ($CD3^{-}$ $GFP^{+}$ cells). Dead cells were excluded from the analysis using 7-AAD live/dead cell exclusion dye. Total cells in the co-culture samples were gated on forward (FSC-A) and side scatter (SSC-A) plots followed by exclusion of cell doublets from the analysis (FSC-H versus FSC-A) and discrimination of live cells (7-AAD negative) versus dead cells (7-AAD positive) via 7-AAD staining (PerCP-Vio700-A channel). Gates were set using biological or FMO controls to identify total effector T cells containing duoCAR-T cells (V1-A::VioBlue-A channel) and $Env^{+}$ $GFP^{+}$ (B1-A::FITC-A channel). Counting beads were used to enumerate absolute cell numbers.

# Supplemental Figure 4

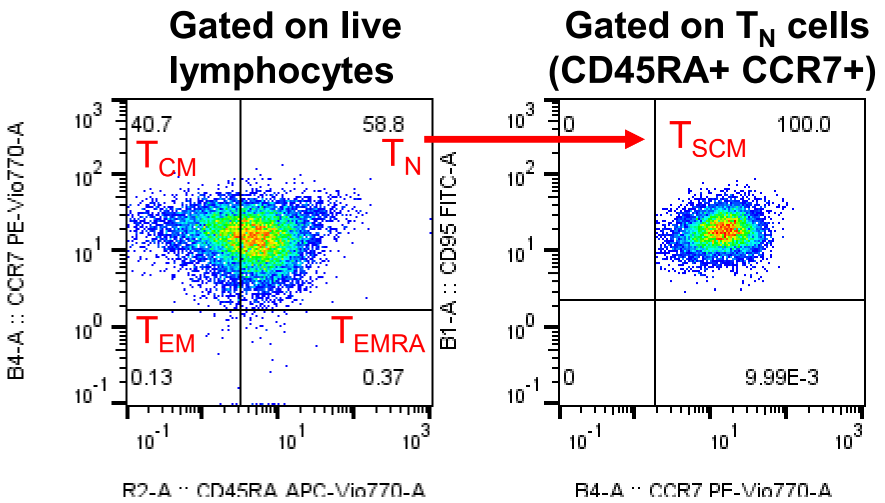


**Supplemental Figure 4. Representative plot of the $T_{SCM}$/$T_{CM}$ anti-HIV duoCAR-T cell memory phenotype**. The pre-infusion anti-HIV duoCAR-T cell product (Day 8, harvest) was stained with an antibody cocktail (T cell memory panel) consisting of APC-Vio770-conjugated anti-CD45RA, PE-Vio770-conjugated anti-CCR7 and FITC-conjugated anti-CD95. Within the live lymphocyte gate (not shown), the T cell memory markers CD45RA, CCR7, and CD95 were used to stratify T cell populations into naive ($T_N$), central memory ($T_{CM}$), effector memory ($T_{EM}$), and effector memory T cells re-expressing CD45RA ($T_{EMRA}$). Early-memory stem cell or stem-cell like ($T_{SCM}$) cells were differentiated from $T_N$ cells based on co-expression of CCR7 and CD95. Anti-HIV duoCAR-T cells were manufactured using the CliniMACS Prodigy (clinical process).

# Supplemental Table 1

**Supplemental Table 1.** Quality and safety profile of research-scale anti-HIV duoCAR-T cell products manufactured from PWH.

| | | | | T CELL PURITY | | | CAR IDENTITY | | | | | |
|---|---|---|---|---|---|---|---|---|---|---|---|---|
| **DONOR ID#** | **HIV Status** | **Visual inspection** | **Viability (%)** | **$CD3^+$ (%)** | **$CD4^+$ (%)** | **$CD8^+$ (%)** | **$CAR^+$ $CD3^+$ (%)** | **$CAR^+$ $CD4^+$ (%)** | **$CAR^+$ $CD8^+$ (%)** | **VCN per cell** | **HIV-1 p24 (pg/mL)** | ***wt* HIV Gag DNA** |
| HGLK 001 | $HIV^+$ | No evidence of contamination | 93.3 | 98.2 | 31.8 | 59.5 | 54.9 | 69.6 | 52.0 | 3.33 | <30 | ND < 1 |
| HGLK 002 | $HIV^+$ | No evidence of contamination | 95 | 98.1 | 35.9 | 45.3 | 36.8 | 63.0 | 47.9 | 1.92 | <30 | ND < 1 |
| HGLK 005 | $HIV^+$ | No evidence of contamination | 90 | 98.1 | 30.1 | 52.5 | 40.1 | 65.2 | 30.4 | 3.49 | <30 | ND < 1 |
| HGLK 022 | $HIV^+$ | No evidence of contamination | 90 | 98.1 | 20.2 | 56.7 | 30.2 | 53.2 | 30.2 | 2.49 | <30 | ND < 1 |
| HGLK 047 | $HIV^+$ | No evidence of contamination | 100 | 98.6 | 18.8 | 53.8 | 49.9 | 75.2 | 66.1 | 3.41 | <30 | ND < 1 |

ND = not detected
